# Frataxin deficiency drives cardiac dysfunction and transcriptional dysregulation in Friedreich ataxia iPSC model

**DOI:** 10.1038/s41419-026-09030-3

**Published:** 2026-06-23

**Authors:** Jarmon G. Lees, Haoxiang Zhang, Lebei Jiao, Anne M. Kong, Ren Jie Phang, Li Li, Nan Su, Sebastian Bass-Stringer, Hei-Yi H. Woo, Anthony S. Mukhtar, Alice Pébay, Mirella Dottori, Louise Corben, Martin Delatycki, Roger Peverill, Stephen Wilcox, Jarny Choi, Jeffrey M. Pullin, Davis McCarthy, Jill S. Napierala, Marek Napierala, Shiang Y. Lim

**Affiliations:** 1 St Vincent’s Institute of Medical Research, Fitzroy, VIC Australia; 2 Department of Medicine and Surgery, University of Melbourne, Parkville, VIC Australia; 3 Drug Discovery Biology, Monash Institute of Pharmaceutical Sciences, Monash University Parkville, Parkville, VIC Australia; 4 Department of Anatomy and Physiology, University of Melbourne, Parkville, VIC Australia; 5 School of Medical, Indigenous, and Health Sciences, Molecular Horizons, Faculty of Science, Medicine and Health, University of Wollongong, Wollongong, NSW Australia; 6 Bruce Lefroy Centre for Genetic Health Research, Murdoch Children’s Research Institute, Parkville, VIC Australia; 7 Department of Paediatrics, Melbourne University, Parkville, VIC Australia; 8 Victorian Clinical Genetics Services, Parkville, VIC Australia; 9 Victorian Heart Institute, Monash University, Clayton, VIC Australia; 10 Walter and Eliza Hall Institute of Medical Research, Parkville, VIC Australia; 11 Melbourne Integrative Genomics, University of Melbourne, Parkville, VIC Australia; 12 Department of Neurology, Peter O’Donnell Jr. Brain Institute, University of Texas Southwestern Medical Center, Dallas, TX USA; 13 National Heart Research Institute Singapore, National Heart Centre, Singapore, Singapore

**Keywords:** Cardiomyopathies, Induced pluripotent stem cells, Stem-cell research, Stem-cell differentiation, Mechanisms of disease

## Abstract

Friedreich ataxia (FRDA) is a progressive neuromuscular degenerative disorder caused by GAA repeat expansions in the *FXN* gene, leading to frataxin deficiency and multisystem pathology. Cardiomyopathy is the leading cause of mortality in individuals with FRDA. To investigate the cellular and molecular mechanisms underlying FRDA-associated cardiac dysfunction, we employed induced pluripotent stem cell (iPSC) lines derived from three individuals with FRDA, each paired with an isogenic control line generated through CRISPR/Cas9-mediated excision of the pathogenic GAA repeat expansion. Correction of the mutation restored *FXN* expression to levels comparable to healthy donor iPSCs, and all lines differentiated efficiently into cardiomyocytes. Functional analysis revealed significant contractile abnormalities in FRDA cardiomyocytes and multicellular cardiac microtissues, including prolonged contraction and relaxation times and faster beating rates, consistent with clinical observations of cardiac contractile dysfunction. FRDA cardiomyocytes also exhibited pathological features such as increased cell size, irregular calcium transients, elevated mitochondrial reactive oxygen species levels, increased mitochondrial fission and increased cell death. These phenotypes were exacerbated by pathological levels of iron supplementation in culture media, highlighting the heightened sensitivity of frataxin-deficient cardiomyocytes to iron-induced metabolic stress. RNA sequencing revealed a distinct transcriptional profile associated with frataxin deficiency. *MEG3* and *PCDHGA10* were consistently dysregulated across all three FRDA-iPSC lines and may represent early molecular markers of FRDA cardiomyopathy. Functional interrogation of these candidates demonstrated that targeted silencing of *MEG3* or *PCDHGA10* in FRDA cardiomyocytes significantly reduced disease‑associated cell death without affecting *FXN* expression. Notably, *PCDHGA10* silencing also normalized elevated mitochondrial reactive oxygen species, whereas *MEG3* silencing did not, highlighting gene‑specific contributions to FRDA cardiomyocyte survival. Collectively, these findings identify *MEG3* and *PCDHGA10* as functionally relevant regulators of FRDA cardiomyocyte pathology.

## Introduction

Friedreich ataxia (FRDA) is one of the most common inherited ataxias, characterized by progressive neurodegeneration and multisystem involvement, primarily affecting the nervous system and the heart [1]. Its prevalence ranges from 1 in 20,000 to 250,000 individuals depending on geography, with symptom onset typically between ages 8 and 15 [2]. Neurological manifestations include progressive limb and gait ataxia, dysarthria, sensory loss, and variable visual and auditory deficits [1], while non-neurological features include scoliosis, diabetes, and cardiomyopathy. The average life expectancy is approximately 35–40 years, with cardiac complications, particularly arrhythmias and congestive heart failure, being the leading causes of mortality [3]. Currently, there are no approved therapies specifically targeting FRDA-associated cardiomyopathy [4].

FRDA results from mutations in the *FXN* gene located on chromosome 9, which encodes frataxin, a mitochondrial protein essential for energy metabolism [5]. Approximately 96% of affected individuals are homozygous for a guanine-adenine-adenine (GAA) trinucleotide repeat expansion in intron 1 of the *FXN* gene [5, 6]. In healthy individuals, the GAA repeat count is typically fewer than 30 repeats, whereas pathogenic expansions exceed 66 repeats, with most individuals with FRDA harboring 600–900 repeats [2]. These expansions induce the formation of repressive heterochromatin, marked by increased histone methylation and reduced acetylation, which suppresses *FXN* transcription and leads to markedly reduced frataxin expression [7].

Frataxin is essential for mitochondrial function, particularly in the biosynthesis of iron-sulfur clusters, which are essential cofactors for multiple mitochondrial enzymes involved in oxidative phosphorylation and energy production [8]. Deficiency of frataxin disrupts the biogenesis of iron-sulfur clusters and mitochondrial iron homeostasis, leading to mitochondrial dysfunction, oxidative stress, and progressive damage to nervous and cardiovascular tissues [9, 10].

Patient-derived induced pluripotent stem cells (iPSCs) carrying the pathogenic GAA expansion have emerged as a robust and genetically accurate platform for modeling FRDA in disease-relevant cell types, particularly cardiomyocytes, overcoming the limitations of immortalized lymphoblastoid lines and primary fibroblasts, which lack the functional characteristics of affected tissues. Since the first FRDA iPSC line was established in 2010 [11], numerous lines have been generated, providing an unlimited supply of patient-specific cells for mechanistic studies and high-throughput screening [12]. FRDA iPSC-derived cardiomyocytes recapitulate key disease features, including reduced frataxin expression, mitochondrial dysfunction, electrophysiological abnormalities, impaired calcium handling, and lipid accumulation, particularly under iron overload [10, 13–18]. These models allow dynamic molecular and functional analyses of tissue-specific disease progression, offering insights into the multifactorial mechanisms driving FRDA cardiomyopathy, including oxidative stress, mitochondrial impairment, and altered iron metabolism. However, limitations remain, including instability of GAA repeats, genotype variability, and the use of unrelated healthy donors as controls, which can introduce confounding genetic differences. Genome editing tools such as CRISPR/Cas9 now enable the generation of isogenic control lines, allowing precise, genetically matched comparisons to overcome these limitations.

In this study, we examined the cellular and molecular characteristics of cardiomyocytes derived from three individuals with FRDA and their corresponding CRISPR/Cas9-corrected isogenic controls. By comparing these paired lines, we aimed to determine how frataxin deficiency affects cardiomyocyte structure, function, and gene expression, and to identify early molecular markers and therapeutic targets of FRDA cardiomyopathy.

## Materials and methods

### Ethics statement

Fibroblasts from three genetically confirmed adults with FRDA were reprogrammed into iPSC lines (FA1–3), and corresponding CRISPR/Cas9-corrected isogenic control lines (FA1ic–3ic) were generated (Table 1). All methods involving human participants were performed in accordance with relevant guidelines and regulations. The protocol for derivation of human primary fibroblasts was approved by the Institutional Review Board (IRB) at the University of Alabama at Birmingham (IRB N131204003). Written informed consent was obtained from all participants prior to study enrollment.

**Table 1:** FRDA patient and iPSC line information.

Individual with FRDA	Sex	GAA1/GAA2 in patient fibroblasts	Derived iPSC lines	Disease state	GAA1/GAA2 in iPSC lines
1	Male	797/797	FA1	FRDA	867/867
			FA1ic	Isogenic control	0/0
2	Male	404/920	FA2	FRDA	550/830
			FA2ic	Isogenic control	0/0
3	Female	367/780	FA3	FRDA	450/450
			FA3ic	Isogenic control	0/0

Fibroblast samples from three confirmed individuals with FRDA were reprogrammed into iPSC lines (FA1-3). The disease-causing GAA repeat expansions were corrected using CRISPR/cas9, generating isogenic control iPSC lines (FA1ic-3ic). At the time of biopsy, Participant 1 exhibited hypertrophic cardiomyopathy; Participant 2 showed a severe cardiac phenotype including reduced ejection fraction, asymmetric septal hypertrophy, and arrhythmia; Participant 3 displayed no detectable cardiac abnormalities.

### Human iPSC lines

Fibroblasts from three genetically confirmed adults with FRDA were reprogrammed into iPSC lines (FA1-3), and corresponding CRISPR/Cas9-corrected isogenic control lines (FA1ic-3ic) were generated as previously described (Table 1) [19]. These iPSC lines were originally derived and characterized by Marek Napierala’s group (University of Texas Southwestern Medical Center) and were recently authenticated by Short Tandem Repeat analysis across 18 loci, with all edited lines matching 100% to their parental lines [20]. Our laboratory received the same authenticated stocks, and all cultures were routinely tested and confirmed mycoplasma‑free. Protocol for derivation of human primary fibroblasts was approved by Institutional Review Board (IRB) at the University of Alabama at Birmingham (IRB N131204003). Both mutant and isogenic iPSC lines were maintained on vitronectin (Thermo Fisher Scientific, MA, USA) coated plates and cultured in StemFlex medium (Thermo Fisher Scientific). A healthy donor iPSC line, CERA007c6 [21], was maintained on vitronectin-coated plates and cultured in TeSR-E8 medium (STEMCELL Technologies, Vancouver, Canada).

### Cardiac microtissue construction

Engineered multicellular cardiac microtissues were constructed according to previously published protocols with modifications [22, 23]. Briefly, day-19 FA1 or FA1ic cardiomyocytes were seeded onto Matrigel-coated 48-well Nunc^TM^ UpCell^TM^ plates (Thermo Fisher Scientific) at a density of 1.2 × 10^5^ cells/cm^2^ (equivalent to 2.0 × 10^5^ cells per cardiac microtissue) in CM replating medium supplemented with 10 μM Y-27632. After 24 h, FA1 or FA1ic iPSC-derived endothelial cells (5.0 × 10^4^ cells/cm^2^), smooth muscle cells (5.0 × 10^3^ cells/cm^2^), autonomic neurons (2.0 × 10^4^ cells/cm^2^), and cardiac fibroblasts (5.0 × 10^3^ cells/cm^2^) were seeded onto the cardiomyocyte layer. The co-culture was maintained in cardiac microtissue medium, composed of a 1:1:1:1:1 ratio of CM medium, endothelial EGM2-MV medium, smooth muscle cell SmGM-2 medium, autonomic neuron medium (DMEM/F12 supplemented with 10% FCS), and cardiac fibroblast FGM-3 medium. This medium was further supplemented with 50 ng/mL VEGF-165, 10 ng/mL BDNF, 10 ng/mL GDNF, 10 ng/mL NGF, and 10 μM Y-27632. After 24 h, the UpCell^TM^ plates were brought to room temperature to release the intact cell sheet, which were then transferred to ultralow attachment plates (Sigma-Aldrich) containing cardiac microtissue medium (without Y-27632) for 24 h. The resulting spheroids were then embedded in 15 µL of growth factor reduced Matrigel and cultured in cardiac microtissue medium (without Y-27632). Cardiac microtissues were maintained in a humidified CO_2_ incubator on an orbital shaker at 60 rpm, with medium changes every 2-3 days.

### Statistics

Sample size was predetermined using a power calculation based on a 10% standard deviation observed in pilot experiments; a sample size of three independent experimental replicates provides >80% power to detect a ≥22% difference using ANOVA (SD = 10%, *α* = 0.05, *β* = 0.2). Data are presented as mean ± standard error of the mean (SEM). Statistical significance was evaluated using either an unpaired Student’s t-test or a two-way ANOVA, followed by Fisher’s Least Significant Difference test for planned pairwise comparisons, as appropriate; tests were two-sided, and data met assumptions for parametric testing with comparable variances across groups. For RNA sequencing, differential expression was assessed using edgeR with Benjamini–Hochberg false discovery rate (FDR) control (FDR < 0.05). Here, n denotes biological replicates (independent differentiations from distinct iPSC lines or independently constructed microtissues); technical replicates (multiple wells or imaging fields) were averaged within each biological replicate. Experiments were replicated in 3–6 independent runs unless otherwise specified, and exact *n* values are reported in the figure legends. Imaging fields were randomly selected for all analyses, and cells were randomly allocated to treatment conditions (e.g., iron exposure, gene knockdown) and to downstream assays. Assessors were blinded to experimental groups where possible. No data were excluded from analyses. A *p*-value < 0.05 was considered statistically significant.

## Results

### Restoration of *FXN* mRNA and FXN protein expression in CRISPR/Cas9-corrected isogenic control iPSCs and cardiomyocytes

**Fig. 1:**
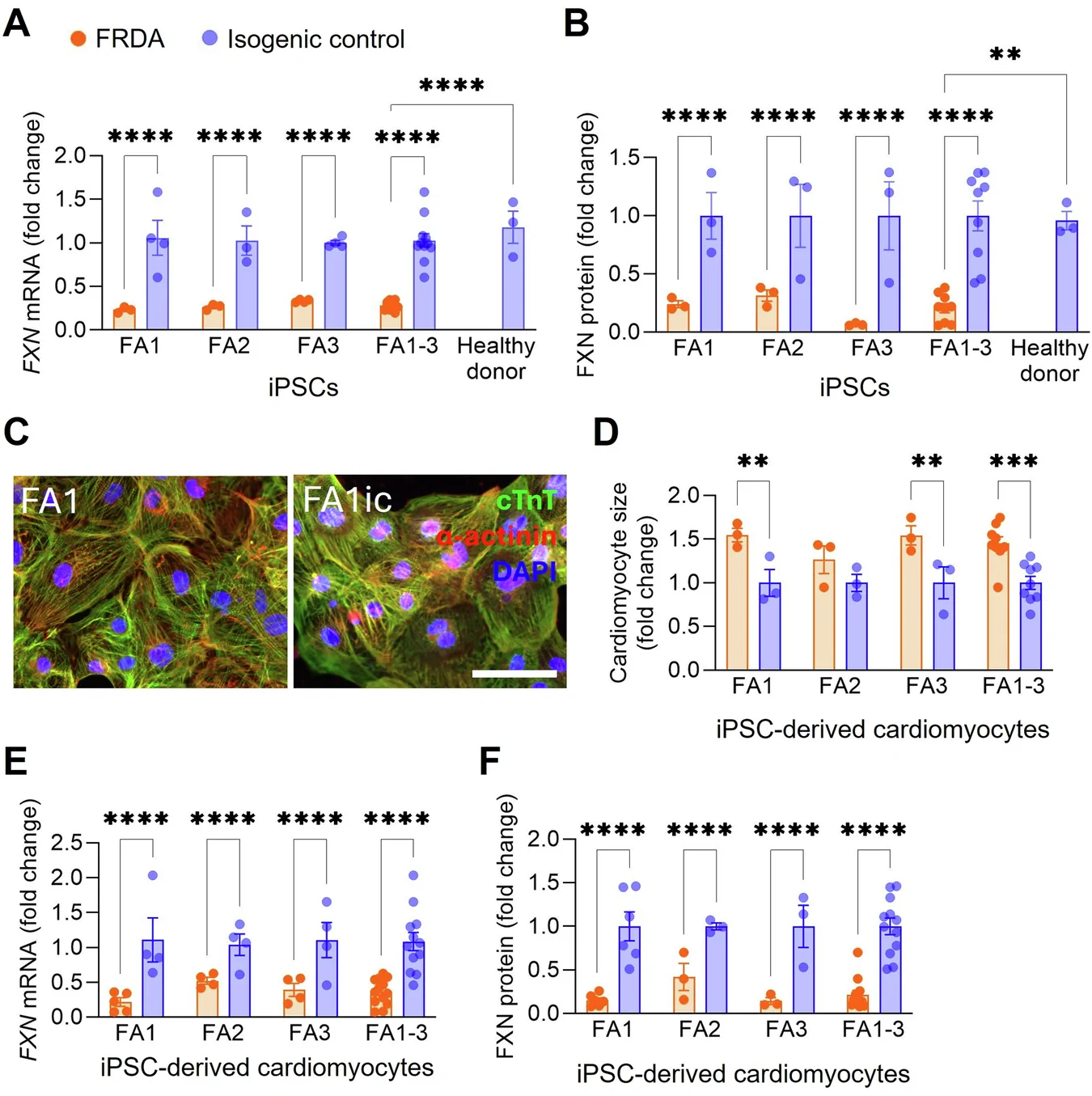
Friedreich ataxia iPSCs and cardiomyocytes exhibit reduced FXN expression compared to isogenic controls. FXN mRNA (**A**) and protein (**B**) expression levels in FRDA patient-derived iPSC lines, isogenic control iPSC lines, and a healthy donor iPSC line (*n* = 3–4 independent experiments). **C** Representative immunofluorescent images of iPSC-derived cardiomyocytes stained with cardiac troponin T (green), α-actinin (red), and DAPI (blue). Scale bar = 50 µm. Quantification of cardiomyocyte size (**D**), *FXN* mRNA expression (**E**), and FXN protein expression (**F**) in FRDA and isogenic control iPSC-derived cardiomyocytes (*n* = 3-6 independent experiments). Data are presented as mean ± SEM for individual iPSC lines (FA1, FA2, FA3) and a pooled average across all 3 lines (FA1-3). **p < 0.01, ***p < 0.001, ****p < 0.0001 by unpaired t-test.

To model FRDA, iPSC lines were established from three individuals with FRDA (FA1–3), along with isogenic controls (FA1ic–3ic) generated via CRISPR/Cas9-mediated excision of the pathogenic GAA repeat expansion (Table 1). Both FRDA and isogenic control iPSC lines displayed morphology typical of pluripotent stem cells, forming flat, compacted colonies with no discernible signs of spontaneous differentiation under pluripotency-maintaining conditions. *NANOG* and *SOX2* mRNAs were detected in all lines, with small line‑specific differences between FRDA and isogenic controls but no consistent pattern across FA1-3 cell lines (Supplementary Fig. 1). Removal of the GAA repeat expansion resulted in a significant increase in FXN mRNA and protein expression across all three isogenic control iPSC lines, restoring levels comparable to those observed in a healthy donor iPSC line (Fig. 1A, B). In addition, excision of the GAA repeats reduced hypermethylation of the FRDA-related differentially methylated regions in the isogenic control iPSCs to levels comparable to unaffected control iPSCs (Supplementary Fig. 2).

Cardiomyocytes were successfully differentiated from both FRDA and isogenic control iPSC lines. Across all lines, cardiomyocytes uniformly expressed cardiac-specific protein markers, including cardiac troponin T and α-actinin (Fig. 1C). At the RNA level, cardiomyocyte markers *TNNT2* and *ACTC1* were consistently upregulated upon differentiation, with no significant differences between FRDA and their matched isogenic controls, indicating comparable acquisition of cardiomyocyte identity across genotypes (Supplementary Fig. 1). A significant increase in cardiomyocyte cell size, suggestive of pathological hypertrophy, was observed in two of the three FRDA lines (Fig. 1D). Importantly, FXN mRNA and protein expression levels in the differentiated cardiomyocytes mirrored those observed in the undifferentiated iPSCs, with isogenic control cardiomyocytes showing significantly higher FXN expression compared to their FRDA counterparts (Fig. 1E, F).

### Friedreich ataxia iPSC-derived cardiomyocytes exhibit contractile dysfunction

**Fig. 2:**
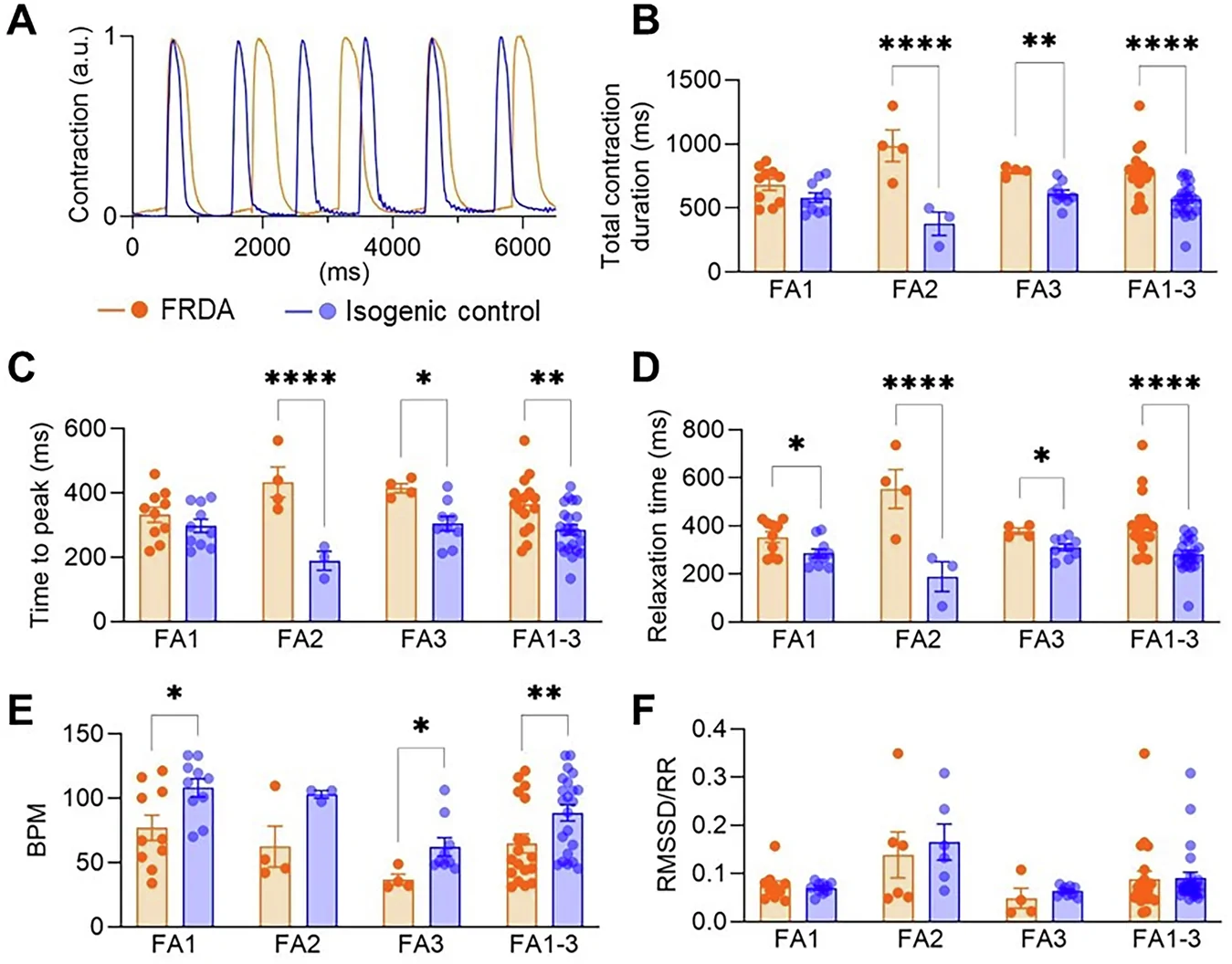
Contractile dysfunction in Friedreich ataxia iPSC-derived cardiomyocytes. **A** Representative contraction trace of FRDA and isogenic control cardiomyocytes (a.u. arbitrary units). Quantification of total contraction duration (**B**), time to peak contraction (**C**), relaxation time (**D**), beating rate (BPM) (**E**), and beat rate variability (RMSSD/RR) (**F**) in FRDA and isogenic control cardiomyocytes (*n* = 3–5 independent experiments). Data represent mean ± SEM for individual iPSC lines (FA1, FA2, FA3) and the pooled average across all 3 lines (FA1-3). *p < 0.05, **p < 0.01, ****p < 0.0001 by unpaired t-test.

To determine whether FRDA iPSC-derived cardiomyocytes recapitulate the cardiac contractile dysfunction observed in individuals with FRDA, contractile function was assessed by measuring total contraction duration (total time taken to contract and relax), time to peak contraction (a surrogate for systolic function), and relaxation time (a surrogate for diastolic function). Compared to their isogenic control cardiomyocytes, FRDA cardiomyocytes from FA2 and FA3 iPSC lines exhibited significantly prolonged total contraction duration, time to peak, and relaxation time (Fig. 2A–D). In contrast, the FA1 cardiomyocytes did not exhibit prolonged total contraction duration or time to peak (Fig. 2B, C), although relaxation time was significantly prolonged (Fig. 2D). When data from all three FRDA lines (FA1-3) were pooled, a significant overall prolongation of all contractile parameters was observed, consistent with impaired systolic and diastolic function. Beating rate (beats per minute, BPM) was significantly lower in the FA1 and FA3 FRDA lines compared to their isogenic controls, but no difference was observed in the FA2 line (Fig. 2E). Beat rate variability, an indicator of arrhythmic risk, did not differ between FRDA and isogenic control cardiomyocytes (Fig. 2F).

### The incidence of irregular cardiomyocyte calcium oscillations is increased in Friedreich ataxia iPSC-derived cardiomyocytes

**Fig. 3:**
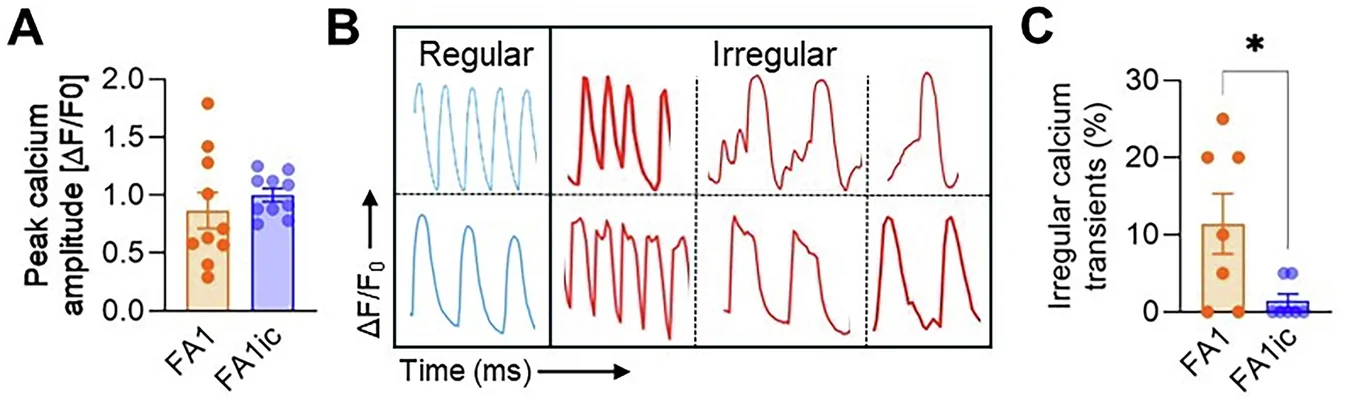
Friedreich ataxia iPSC-derived cardiomyocytes exhibit a high frequency of irregular calcium transients. **A** Peak calcium amplitude of FRDA and isogenic control iPSC-derived cardiomyocytes (*n* = 3-6 independent experiments). **B** Representative traces of cardiomyocyte calcium cycling. Calcium transients were categorized into either regular or irregular. **C** Percentage of cardiomyocytes exhibiting irregular calcium transients (*n* = 3-6 independent experiments). Data are presented as mean ± SEM. *p < 0.05, **p < 0.01 by unpaired t-test.

Calcium signaling plays a critical role in the contraction and relaxation of cardiomyocytes [24]. In individuals with FRDA, abnormalities in calcium signaling, both in intensity and oscillation stability, have been reported and are associated with arrythmias and sudden cardiac death [25]. Calcium dynamics in cardiomyocytes were assessed using fluorescence microscopy and Fluo-4 calcium indicator. The maximum change in intracellular calcium levels did not differ significantly between FRDA cardiomyocytes and their isogenic controls (Fig. 3A). However, distinct differences were observed in the regularity of calcium transients. These were categorized as either regular or irregular according to established guidelines [26, 27] (Fig. 3B). FRDA cardiomyocytes exhibited a significantly higher incidence of irregular calcium oscillations compared to isogenic control cardiomyocytes.

To further evaluate the arrhythmic potential of FRDA-iPSCs derived cardiomyocytes, multielectrode array recording was performed to measure the extracellular field potential of 2D cultured cardiomyocytes (Supplementary Fig. 3A, B). FRDA cardiomyocytes exhibited a significantly longer field potential duration compared to isogenic controls; however, this difference was no longer significant after correction for beating frequency (Supplementary Fig. 1C, D).

### Friedreich ataxia iPSC-derived cardiomyocytes exhibit mitochondrial dysfunction

**Fig. 4:**
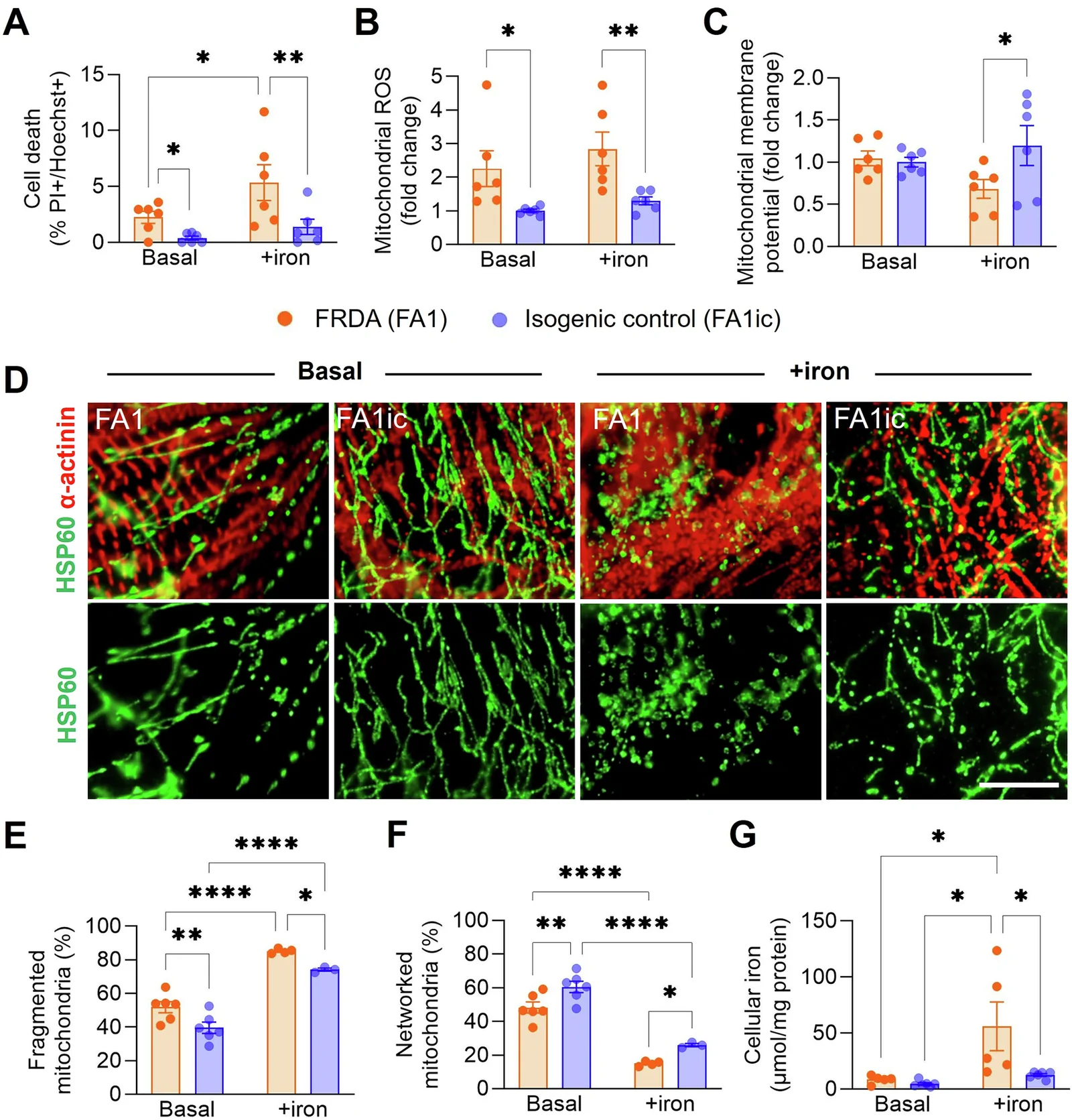
Mitochondrial dysfunction in Friedreich ataxia iPSC-derived cardiomyocytes is exacerbated by iron-induced metabolic stress. **A** Percentage of cell death, **B** mitochondrial reactive oxygen species (ROS) levels, **C** mitochondrial membrane potential, **D** representative images of cardiomyocyte mitochondria stained for Hsp60 (green) and α-actinin (red) (scale bar = 10 µm); quantification of the percentage of cells with fragmented (**E**) and networked (**F**) mitochondria, and (**G**) cellular iron content (µmol/mg protein) in cardiomyocytes derived from FRDA (FA1) and isogenic control (FA1ic) iPSCs. Cells were cultured in CM medium under basal conditions or supplemented with 200 µM iron sulfate for 48 h (+iron) (*n* = 3 independent experiments). Data are presented as mean ± SEM. *p < 0.05, **p < 0.01, ****p < 0.0001 by two-way ANOVA followed by Fisher’s LSD test for planned pairwise comparisons.

Frataxin is a mitochondrial protein essential for maintaining mitochondrial homeostasis and function, with critical implications for cardiac health. We next evaluated whether FRDA-iPSC-derived cardiomyocytes exhibited the mitochondrial dysfunction characteristic of FRDA. Cardiomyocytes derived from the FA1 iPSC line exhibited a significant increase in cell death compared to their isogenic control (FA1ic) counterparts (Fig. 4A and Supplementary Fig. 4), along with an approximately 2-fold increase in mitochondrial reactive oxygen species (ROS) levels (Fig. 4B). Mitochondrial membrane potential was similar between FRDA and isogenic control cardiomyocytes (Fig. 4C). When mitochondrial morphology was categorized as either predominantly networked or fragmented (Fig. 4D), FA1 FRDA cardiomyocytes displayed a clear shift from predominantly networked to fragmented mitochondrial morphology compared to their FA1ic isogenic controls (Fig. 4E, F).

Deficiency in iron-sulfur cluster biogenesis, a hallmark of frataxin loss, compromises mitochondrial function and can lead to depolarization of the mitochondrial membrane potential, particularly under conditions of iron-induced metabolic stress, This phenomenon has been previously noted in mouse cerebellar granule cells [28] and FXN-silenced human neuroblastoma cells [29]. To determine whether this phenotype could be recapitulated in iPSC-derived cardiomyocytes, a disease-relevant cellular stressor was applied. Elevated iron levels have been reported in the left ventricle of individuals with FDRA [30], and abnormal iron aggregates have been detected throughout the myocardium [31], indicating iron dysregulation. This can be modeled in vitro through iron supplementation in culture [16, 17]. Baseline cellular iron content did not differ significantly between FRDA and control cardiomyocytes under basal culture conditions. However, iron supplementation (200 µM iron sulfate for 48 h) significantly increased cellular iron levels in FRDA cardiomyocytes compared with iron-treated control cells as well as both basal groups (Fig. 4G). Treatment with iron sulfate for 48 h also resulted in significantly increased cell death in FRDA iPSC-derived cardiomyocytes, without notably affecting the viability of isogenic control cardiomyocytes (Fig. 4A and Supplementary Fig. 4). While iron supplementation did not further elevate mitochondrial ROS levels in FRDA cardiomyocytes, which already exhibited higher ROS compared to controls (Fig. 4B), it revealed a difference in mitochondrial membrane potential, with FRDA cardiomyocytes showing significantly lower mitochondrial membrane potential than their isogenic counterparts (Fig. 4C). Additionally, iron-induced metabolic stress increased mitochondrial fragmentation in both FRDA and control cardiomyocytes, indicating a generalized mitochondrial stress response (Fig. 4D–F).

### Friedreich ataxia iPSC-derived multicellular cardiac microtissues exhibit cellular injury and contractile abnormalities

**Fig. 5:**
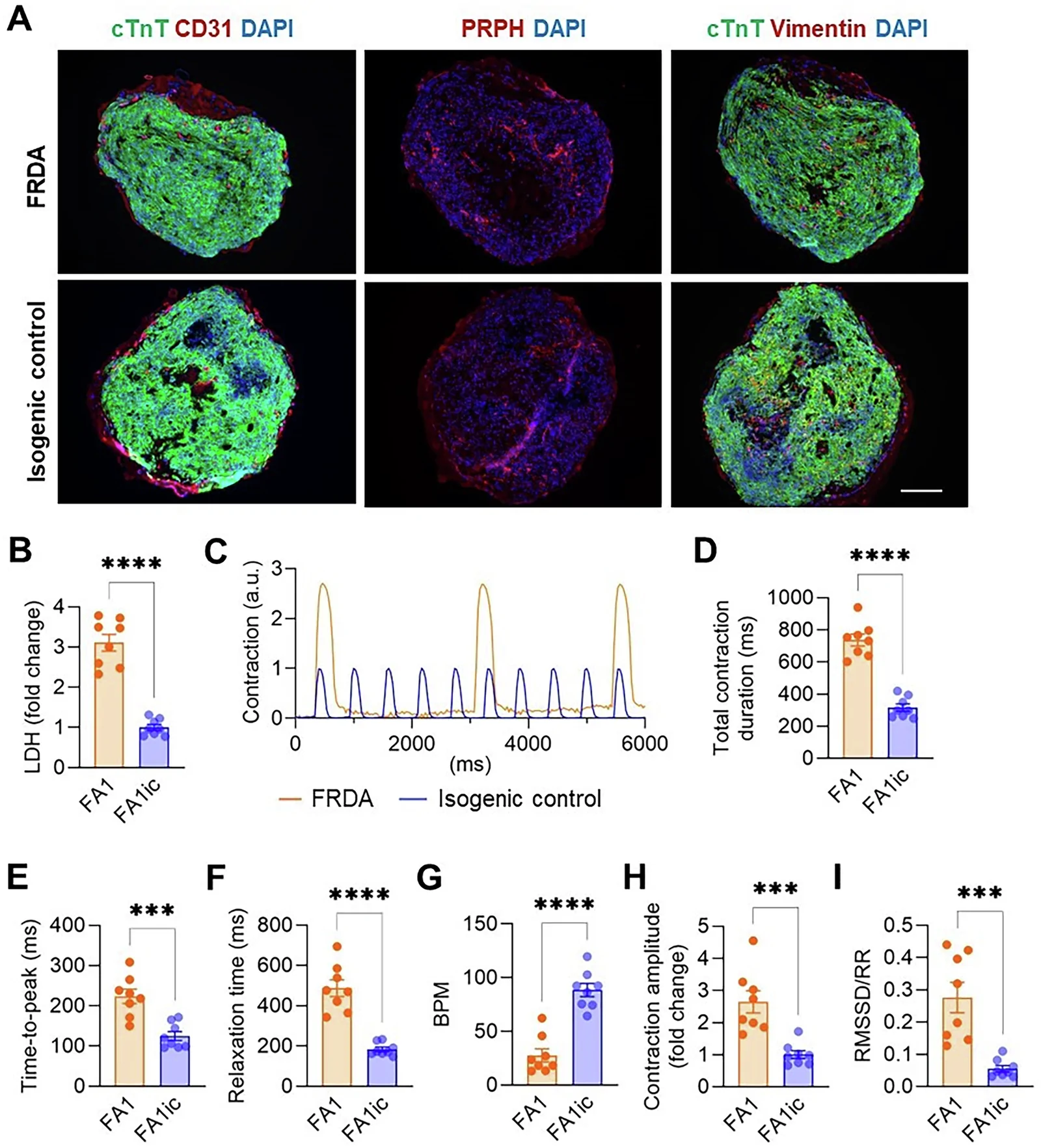
Friedreich ataxia iPSC-derived multicellular cardiac microtissues exhibit cardiac dysfunction. **A** Representative images of multicellular cardiac microtissues engineered from FRDA (FA1) or isogenic control (FA1ic) iPSCs, stained for cardiac troponin T positive cardiomyocytes (cTnT, green), CD31 positive endothelial cells (red), peripherin positive autonomic neurons (PRPH, red), and vimentin positive cardiac fibroblasts (red). Scale bar = 200 µm. **B** Lactate dehydrogenase (LDH) release in FA1 and FA1ic multicellular cardiac microtissues (*n* = 4 independent experiments). **C** Representative contraction trace of FA1 and FA1ic cardiac microtissues (a.u. arbitrary units). Quantification of contraction parameters: total contraction time (**D**), time to peak contraction (**E**), relaxation time (**F**), beating rate (BPM) (**G**), peak contraction amplitude (**H**), and beat rate variability (RMSSD/RR) (**I**) (*n* = 4 independent experiments). Data are presented as mean ± SEM Data are mean ± SEM. ***p < 0.001, ****p < 0.0001 by unpaired t-test.

A multicellular cardiac microtissue model, composed of cardiomyocytes along with non-cardiomyocyte populations such as endothelial cells, smooth muscle cells, autonomic neurons and cardiac fibroblasts (Fig. 5A), was employed to better recapitulate the 3D microenvironment and heterogeneous cellular composition of native heart tissues. FRDA iPSC-derived cardiac microtissues showed elevated levels of lactate dehydrogenase in the conditioned media compared to isogenic control-derived microtissues, indicating increased cellular injury (Fig. 5B). Consistent with findings in 2D cultures, FRDA cardiac microtissues also demonstrated contractile abnormalities (Fig. 5C), including prolonged total contraction duration (Fig. 5D), time to peak contraction (Fig. 5E), and relaxation time (Fig. 5F), as well as a reduced beating rate (Fig. 5G) relative to isogenic controls. Interestingly, FRDA microtissues exhibited a higher contraction amplitude compared to controls (Fig. 5H). Moreover, beat rate variability was significantly increased in FRDA iPSC-derived microtissues (Fig. 5I), a feature not observed in the 2D cardiomyocyte model (Fig. 2F).

### Transcriptomic changes in Friedreich ataxia iPSC-derived cardiomyocytes

**Fig. 6:**
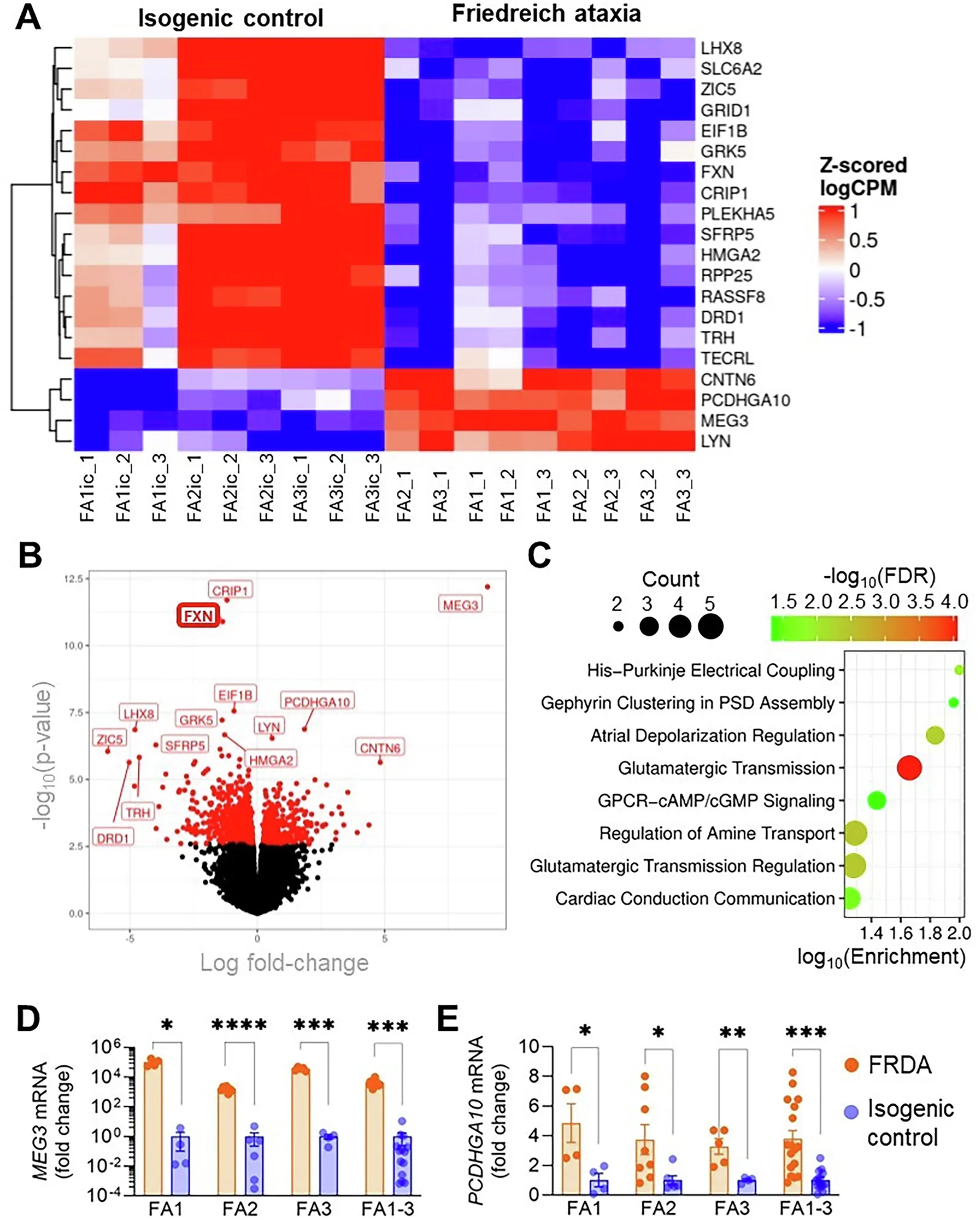
Transcriptomic analysis of cardiomyocytes derived from Friedreich ataxia and isogenic control iPSCs. **A** Heatmap of globally identified differentially expressed genes in cardiomyocytes derived from all FRDA and isogenic control iPSC lines. **B** Volcano plot showing differentially expressed genes from a combined analysis of cardiomyocytes from all three iPSC lines versus isogenic controls (FA1-3 versus FA1ic-3ic). Genes in red have an adjusted *p*-value (FDR) < 0.05. **C** GO Biological Processes identified using the PANTHER Overrepresentation Test, applied to differentially expressed genes with a false discovery rate <0.05 and an absolute log fold change >2. The top eight enriched biological processes are shown. RT-qPCR analysis of *MEG3* (**D**) and *PCDHGA10* (**E**) mRNA expression in iPSC-derived cardiomyocytes. Data represent mean ± SEM for individual iPSC lines (FA1, FA2, FA3) and the pooled average across all 3 lines (FA1-3). *p < 0.05, **p < 0.01, ***p < 0.001, ****p < 0.0001 by unpaired t-test.

To investigate the transcriptional consequences of frataxin deficiency in cardiomyocytes, bulk RNA sequencing was performed on iPSC-derived cardiomyocytes from three FRDA iPSC lines (FA1–FA3) and their isogenic control iPSC lines (FA1ic–FA3ic) (Fig. 6A–C). This approach allowed for both patient-specific comparisons and pooled analysis to identify differentially expressed genes (DEGs) associated with the FRDA cardiac phenotype. Each patient line was analyzed independently against its matched isogenic control to account for individual genetic backgrounds.

The number of DEGs varied considerably across patient lines, with 13, 2445, and 5280 DEGs identified in FA1, FA2, and FA3 respectively (Supplementary Tables 1–3). Of note, *MEG3* (maternally expressed gene 3) and *PCDHGA10* (protocadherin gamma subfamily A-10) were consistently dysregulated across all three FRDA patient lines, suggesting their potential as core transcriptional markers of FRDA cardiomyopathy (Fig. 6A–C and Supplementary Table 4). *MEG3*, a long non-coding RNA involved in apoptosis and oxidative stress regulation, was the most significantly upregulated gene in FRDA cardiomyocytes. *PCDHGA10*, a calcium-dependent cell adhesion molecule, was upregulated and has known roles in neuronal circuit formation and emerging relevance in cardiac tissue organization and remodeling. *MEG3* and *PCDHGA10* expression levels were further validated by qPCR (Fig. 6D, E). Pooled analysis of all FRDA samples confirmed a significant downregulation of *FXN* expression in FRDA iPSC-derived cardiomyocytes (Fig. 6A, B) (Table 2). Additional DEGs were identified, many of which are implicated in calcium dysregulation (e.g., *TECRL*, *TRH*, *SLC6A2*, *GRID1*), contractile and electrophysiological dysfunction (e.g., *ERK5*, *LYN*, *LHX8*, *PCDHGA10*, *CNTN6*, *PLEKHA5*, *RASSF8*), mitochondrial dysfunction and oxidative stress (e.g., *MEG3*, *EiF1B*, *HMGA2*, *ZIC5*), and cellular stress and apoptosis (e.g., *CRIP1*, *RPP25*, *SFRP5*, *HMGA2*, *ZIC5*) (Table 2), consistent with enrichment of GO biological processes related to synaptic signaling, cardiac conduction, and ion transport (Fig. 6C and Supplementary Table 5). Collectively, these transcriptomic changes provide insight into the potential molecular mechanisms underlying FRDA cardiomyopathy and identify candidate genes for further functional studies and potential therapeutic targeting.

**Table 2:** Top 15 differentially expressed genes in FRDA-iPSC-derived cardiomyocytes from three individuals with FRDA, ranked by false discover rate.

Gene name	Adjusted *p*-value	Log fold-change
*MEG3*	1.07e-8	9.03
*CRIP1*	1.78e-8	−1.19
*FXN*	6.55e-8	−1.36
*EIF1B*	0.000119	−0.915
*GRK5*	0.000206	−1.38
*LHX8*	0.000338	−4.8
*PCDHGA10*	0.000338	1.85
*HMGA2*	0.000464	−1.28
*LYN*	0.000551	0.584
*SFRP5*	0.000882	−3.98
*TECRL*	0.00115	−1.46
*ZIC5*	0.00129	−5.87
*RASSF8*	0.00163	−1.39
*RPP25*	0.00163	−1.12
*TRH*	0.0017	−4.63

Negative log fold change values indicate downregulation in FRDA-iPSC-derived cardiomyocytes relative to their corrected isogenic controls.

### Silencing of *MEG3* or *PCDHGA10* alleviates cardiomyocyte death in Friedreich ataxia

Given the consistent upregulation of *MEG3* and *PCDHGA10* in cardiomyocytes derived from three independent FRDA iPSC lines, we next investigated whether these transcripts contribute functionally to the FRDA cardiomyocyte phenotype. Targeted knockdown experiments were performed in FA1 FRDA iPSC-derived cardiomyocytes using antisense oligonucleotide (ASO) GapmeRs to silence the long non‑coding RNA *MEG3*, and a SMARTpool siRNA approach to silence the protein‑coding gene *PCDHGA10*.

**Fig. 7:**
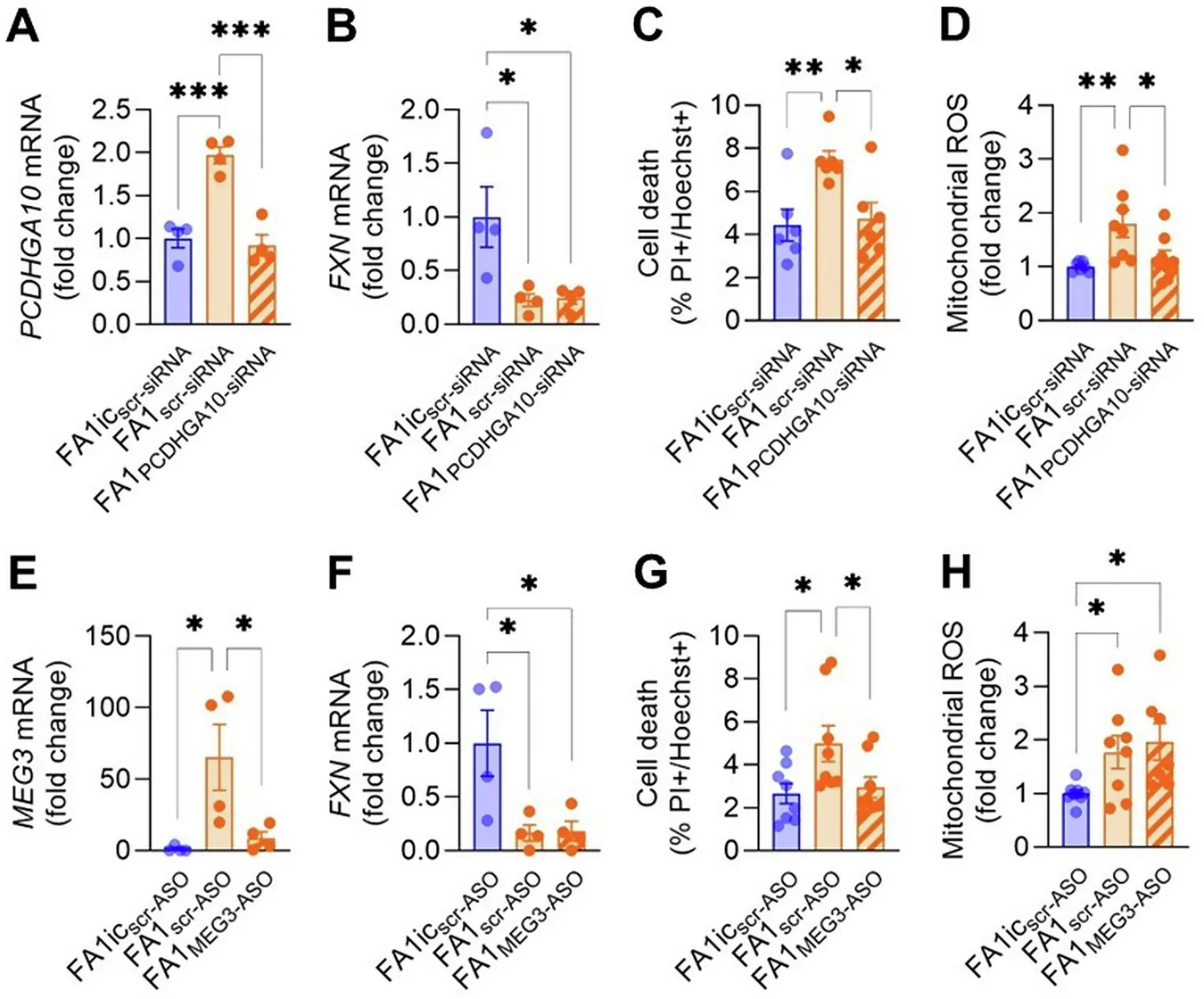
Silencing of *PCDHGA10* or *MEG3* improves cardiomyocyte survival in FRDA. **A**–**D** SMARTPool siRNA‑mediated knockdown of *PCDHGA10* in cardiomyocytes derived from FRDA (FA1) and isogenic control (FA1ic) iPSC lines, with scrambled siRNA included in both groups. **A** *PCDHGA10* mRNA expression, **B** *FXN* mRNA expression, **C** cell death assessed by propidium iodide (PI) and Hoechst staining, and **D** mitochondrial reactive oxygen species (ROS). **E**–**H** Antisense oligonucleotide (ASO)‑mediated knockdown of the long non-coding RNA *MEG3* in the same model, with scrambled ASO controls. **E** *MEG3* expression, **F** *FXN* mRNA expression, **G** cell death assessed by PI+Hoechst staining, and **H** mitochondrial ROS. *n* = 4 independent experiments. Data are presented as mean ± SEM. *p < 0.05, **p < 0.01, ***p < 0.001 by one-way ANOVA followed by Fisher’s LSD test.

*PCDHGA10* knockdown efficiently reduced its mRNA expression (Fig. 7A) without altering *FXN* mRNA levels, which remained significantly reduced relative to isogenic controls (Fig. 7B). Functionally, FRDA cardiomyocytes exhibited approximately two‑fold higher cell death compared to controls, which was significantly reduced to near-control levels following *PCDHGA10* silencing (Fig. 7C and Supplementary Fig. 5A). In parallel, elevated mitochondrial ROS levels in FRDA cardiomyocytes were also restored to control levels upon *PCDHGA10* knockdown (Fig. 7D).

A similar approach was used to investigate the role of *MEG3*. ASO‑mediated silencing significantly reduced *MEG3* transcript levels in FRDA cardiomyocytes to those comparable with isogenic controls (Fig. 7E), without affecting *FXN* mRNA expression (Fig. 7F). *MEG3* knockdown likewise significantly reduced cardiomyocyte death to control levels (Fig. 7G and Supplementary Fig. 5B). However, in contrast to *PCDHGA10* knockdown, *MEG3* silencing did not reduce mitochondrial ROS levels, which remained significantly elevated compared to isogenic controls (Fig. 7H). Collectively, these findings demonstrate that both *MEG3* and *PCDHGA10* contribute to cardiomyocyte loss in FRDA, likely acting downstream of frataxin deficiency, while exerting distinct effects on mitochondrial oxidative stress.

## Discussion

This study presents the development and characterization of a human iPSC-based model of FRDA cardiomyopathy, using three patient-derived iPSC lines and their CRISPR/Cas9-corrected isogenic controls. Restoration of *FXN* expression through targeted genome editing enabled the isolation of the effects of frataxin deficiency on cardiomyocyte function and gene regulation. The model revealed early and intrinsic cardiac abnormalities in FRDA, including contractile dysfunction, impaired calcium handling, compromised viability, mitochondrial oxidative stress, mitochondrial fission, and transcriptional dysregulation. Mitochondrial disease phenotypes were further exacerbated under iron-induced metabolic stress conditions, highlighting the metabolic vulnerability of FRDA cardiomyocytes. Transcriptomic analysis identified a distinct molecular signature, with *MEG3* and *PCDHGA10* consistently dysregulated across all FRDA lines. Importantly, targeted silencing of either *MEG3* or *PCDHGA10* in FRDA cardiomyocytes rescued disease‑associated cell death. Notably, only *PCDHGA10* silencing additionally normalized elevated mitochondrial ROS, indicating gene‑specific contributions to cardiomyocyte pathology. Together, these results provide mechanistic insight into the early stages of FRDA cardiomyopathy and identify *MEG3* and *PCDHGA10* as functionally relevant downstream drivers and potential therapeutic targets.

Cardiac manifestations are a primary cause of mortality in FRDA, with structural remodeling and arrhythmias often evident early in disease progression [32]. Despite this, the underlying cellular mechanisms remain incompletely understood [33]. Previous studies using FRDA iPSC-derived cardiomyocytes have reported abnormalities in contractility and calcium handling [14, 34]; however, the absence of isogenic controls in those studies limited the ability to attribute observed phenotypes specifically to *FXN* deficiency. In vitro modeling of FRDA is challenged by inter-patient genetic variability and phenotypic heterogeneity. This limitation was addressed in the present study through CRISPR/Cas9-mediated excision of the pathogenic GAA repeat expansion in the *FXN* gene, which restored frataxin mRNA and protein levels and reversed multiple contractile and mitochondrial abnormalities. Although this correction yields a genetic state not typically found in healthy individuals, who usually carry 8 to 30 GAA repeats [35], the resulting isogenic control iPSCs expressed frataxin at levels comparable to healthy donor-derived iPSCs, supporting their validity as physiologically relevant controls.

Genetically matched control lines enables precise characterization of FRDA-associated cardiac phenotypes. Across multiple FRDA lines, prolonged contraction and relaxation times point to intrinsic systolic and diastolic defects caused by frataxin deficiency. The prolonged time-to-peak contraction observed in FRDA cardiomyocytes parallels findings in failing human myocardium, underscoring the pathological relevance of our model for modeling early-stage FRDA cardiomyopathy [36]. In 3D cardiac microtissues, FRDA microtissues showed prolonged contraction duration and increased amplitude despite reduced beat rate. This pattern contrasts with the typical force-frequency relationship observed in adult cardiomyocytes, where lower beating rates are generally associated with a reduced contraction amplitude [36]. This discrepancy may reflect the immaturity of iPSC-derived cardiomyocytes, the absence of physiological loading and pacing, and the lack of systemic neurohumoral regulation in vitro.

Heart rate is a critical variable in FRDA pathology, particularly when interpreting systolic function [37]. While our FRDA models consistently exhibited lower BPM, in line with a previous study using FRDA iPSC-derived cardiomyocytes [14], clinical studies report elevated heart rates in individuals with FRDA and preserved left ventricular ejection fraction, who nonetheless show impaired systolic and early diastolic velocities [38, 39]. Similarly, frataxin knockout mice exhibit bradycardia and reduced contraction amplitude, with only partial recovery of heart rate after frataxin restoration but persistent contractile impairment [40]. Together, these findings suggest that frataxin deficiency induces intrinsic, partly irreversible contractile defects, shaped by both cell-autonomous and systemic factors.

Aberrant calcium handling is a key pathological feature in FRDA cardiomyocytes. Although calcium transient amplitude and beat rate variability remained unchanged in 2D monolayer cultures, FRDA iPSC-derived cardiomyocytes displayed irregular calcium oscillations, indicating disrupted excitation–contraction coupling. Interestingly, the lack of changes in calcium transient amplitude contrasts with a previous study reporting reduced calcium transient amplitude in FRDA iPSC-derived cardiomyocytes compared to human ESC-derived cardiomyocytes (rather than isogenic controls) [14], suggesting that baseline calcium handling may be influenced by both the choice of control cells and differences in genetic background. In 3D multicellular cardiac microtissues, the increased beat rate variability indicates that interactions within a complex multicellular environment amplify susceptibility to arrhythmias. Non-cardiomyocyte populations, such as cardiac fibroblasts and autonomic neurons, may contribute to this electrical instability, consistent with clinical reports of conduction defects and arrhythmias in FRDA [33]. Mechanistically, frataxin deficiency has been linked to impaired mitochondrial calcium uptake and ATP production, compromising calcium-handling proteins such as SERCA and the sodium/calcium exchanger [14, 29]. This dysfunction in turn promotes cytosolic sodium accumulation, reversal of exchanger activity, and increased ROS, which further destabilize calcium channels and promote arrhythmogenesis [41]. Supporting systemic involvement, FRDA fibroblasts show downregulation of calcium exchanger genes, indicating broader calcium dysregulation beyond cardiomyocytes [42].

Frataxin, a mitochondrial protein essential for iron sulfur cluster biogenesis and mitochondrial homeostasis, regulates oxidative metabolism and redox balance [43]. Its deficiency in FRDA impairs mitochondrial function, elevates ROS, and reduces mitochondrial membrane potential in yeast, animal models, and human cell lines [5, 44, 45]. In the current model, FRDA iPSC-derived cardiomyocytes exhibited increased mitochondrial ROS levels, elevated cell death, and a shift toward fragmented mitochondrial morphology, consistent with the established role of frataxin in maintaining redox balance and mitochondrial integrity [9, 18, 29]. Despite these abnormalities, baseline mitochondrial membrane potential remained unchanged, as reported in primary fibroblasts isolated from individuals with FRDA [46]. This may suggest the activation of compensatory mechanisms during early cardiomyocyte development or the presence of a threshold that must be exceeded to trigger depolarization. To test this, iron overload, a disease-relevant stressor that mimics metabolic stress caused by myocardial iron accumulation FRDA [30, 31], was applied. Under these conditions, cellular iron accumulation, mitochondrial membrane depolarization, cell death and mitochondrial fragmentation were significantly exacerbated, with FRDA cardiomyocytes consistently exhibiting more severe phenotypes. Similar results were reported by Lee and colleagues, who found that iron supplementation aggravated calcium dysregulation and cardiac hypertrophy in FRDA iPSC-derived cardiomyocytes [16, 17]. Collectively, these findings suggest that frataxin-deficient cardiomyocytes are predisposed to mitochondrial dysfunction, with iron dysregulation acting as a catalyst for disease progression.

Beyond mitochondrial stress, early pathological remodeling was evident, including cardiomyocyte hypertrophy and increased apoptosis. Cardiac structural remodeling is a hallmark of FRDA cardiomyopathy and has been reported in both iPSC-derived cardiomyocytes and transgenic mouse models [13, 47]. The increased cardiomyocyte size observed in the present study reflects this hypertrophic phenotype, consistent with previous findings in FRDA iPSC-derived cardiomyocytes [6, 16] and with clinical observations of ventricular wall thickening in individuals with FRDA. Nonetheless, this cellular enlargement likely reflects only a subset of the complex structural remodeling observed in the FRDA heart in vivo [48]. The concurrent increase in cell death aligns with autopsy findings of cardiomyocyte loss in FRDA hearts [30], supporting a model in which cumulative cell loss contributes to progressive cardiac remodeling and contractile dysfunction [12].

RNA sequencing was performed to further elucidate the molecular basis of these phenotypes observed in FRDA iPSC-derived cardiomyocytes. Both individual and pooled analyses revealed broad transcriptional changes associated with frataxin deficiency. Pooled analysis identified dysregulation of genes involved in calcium signaling, cardiac rhythm, and hypertrophy, including *CRIP1* (calcium binding and cytoskeletal organization) [49], *GRK5* (β-adrenergic signaling and hypertrophy) [50], and *TECRL* (arrhythmia and calcium handling) [51]. GO enrichment analysis showed overrepresentation of processes related to cardiac conduction, synaptic signaling, and membrane depolarization, pathways critical for coordinated electrophysiological activity and neuro-cardiac integration. These changes align with the functional abnormalities observed in FRDA cardiomyocytes and underscore the widespread impact of reduced frataxin.

Among the DEGs, *MEG3* and *PCDHGA10* were consistently dysregulated in cardiomyocytes derived from three independent FRDA iPSC lines, suggesting they represent core components of the FRDA cardiac transcriptional signature. To determine whether these transcripts contribute functionally to FRDA cardiomyocyte pathology, targeted knockdown of each gene was performed and downstream disease-relevant phenotypes were assessed. *MEG3*, the most significantly upregulated transcript, encodes a long non-coding RNA previously implicated in apoptosis, angiogenesis, and ferroptosis [52, 53]. Consistent with these roles, ASO‑mediated silencing of *MEG3* significantly reduced FRDA cardiomyocyte death to levels comparable to isogenic controls, without affecting *FXN* expression. This finding supports a model in which *MEG3* likely acts downstream of frataxin deficiency to promote cardiomyocyte loss. While prior studies have shown that *MEG3* downregulation is protective in myocardial infarction-induced apoptosis [54] and fibroblast-driven fibrosis [55], our data extend these observations to FRDA by demonstrating a cell‑autonomous, disease‑context‑specific role in cardiomyocyte survival. Notably, *MEG3* silencing did not attenuate elevated mitochondrial ROS levels, indicating that its pro‑death effects are mediated through pathways independent of mitochondrial redox stress. *MEG3* is also the second long non-coding RNA implicated in FRDA, following *TUG1*, which is downregulated in patient blood and serum [56]. While *TUG1* has been proposed as a circulating biomarker, the functional impact of *MEG3* within cardiomyocytes points to a more tissue-specific role in FRDA cardiomyopathy.

*PCDHGA10*, a calcium-dependent cell adhesion molecule, was also significantly upregulated in FRDA cardiomyocytes. *PCDHGA10* is primarily associated with neuronal circuit formation and maintenance [57], and its dysregulation has been linked to neurodegeneration in Huntington’s disease [58] and proprioceptive neuron loss in FRDA [59]. Here, siRNA‑mediated silencing of *PCDHGA10* restored cardiomyocyte survival and significantly reduced elevated mitochondrial ROS levels, without altering *FXN* expression. Transcriptomic evidence has also associated increased *PCDHGA10* expression with disrupted muscle fiber organization and impaired contractility in cardiac contexts [60]. Together, these findings implicate *PCDHGA10* in pathways linking intercellular signaling to mitochondrial dysfunction and oxidative stress in FRDA cardiomyocytes. Its upregulation in FRDA cardiomyocytes may therefore contribute to structural disorganization, impaired intracellular communication, and increased susceptibility to oxidative injury. The differential effects of *MEG3* and *PCDHGA10* silencing on mitochondrial ROS suggest that these genes influence cardiomyocyte survival through distinct mechanisms. Collectively, the dysregulation of *MEG3* and *PCDHGA10* highlights key processes underlying FRDA cardiomyopathy. Importantly, their functional validation extends their relevance beyond transcriptomic markers to candidate downstream effectors of cardiomyocyte injury, underscoring the value of isogenic iPSC models for identifying and testing disease‑relevant therapeutic targets.

## Conclusion

This study provides a comprehensive characterization of cardiomyocyte pathology in FRDA using patient-derived iPSC models alongside CRISPR/cas9-corrected isogenic controls. Frataxin deficiency was found to cause early and intrinsic cardiomyocyte dysfunction, characterized by impaired contractility, irregular calcium handling, elevated mitochondrial oxidative stress, mitochondrial fission, and increased cell death. These pathogenic phenotypes were evident under baseline conditions and were further exacerbated by iron-induced metabolic stress conditions. Transcriptomic profiling revealed a distinct molecular signature associated with *FXN* downregulation, with *MEG3* and *PCDHGA10* emerging as consistently dysregulated genes across all patient lines. Importantly, functional silencing of *MEG3* or *PCDHGA10* rescued cardiomyocyte survival, establishing these transcripts as active contributors to FRDA cardiomyocyte pathology rather than passive disease markers.

The use of genetically matched isogenic iPSC models enabled precise dissection of disease-specific phenotypes and highlights their utility as a platform for uncovering pathogenic mechanisms and guiding the development of targeted therapeutic strategies.

## Supplementary information

## Data Availability

The Gene Expression Omnibus accession number for the RNA-seq dataset reported in this paper is GSE305638.
