# Supplementary material for: Frataxin deficiency drives cardiac dysfunction and transcriptional dysregulation in Friedreich ataxia iPSC model
Source: Cell Death Dis. 2026 Jun 23;17(1):774. doi: 10.1038/s41419-026-09030-3 (PMC13538398; doi:10.1038/s41419-026-09030-3)
Supplement: Supplementary file 2 [file 41419_2026_9030_MOESM1_ESM.docx]

**Supplementary Figures and Legends**

**Frataxin deficiency drives cardiac dysfunction and transcriptional dysregulation in Friedreich ataxia iPSC model**

**Materials and methods**

**Human iPSC lines**

Fibroblasts from three genetically confirmed adults with FRDA were reprogrammed into iPSC lines (FA1-3), and corresponding CRISPR/Cas9-corrected isogenic control lines (FA1ic-3ic) were generated as previously described (1) (Table 1). Protocol for derivation of human primary fibroblasts was approved by Institutional Review Board (IRB) at the University of Alabama at Birmingham (IRB N131204003). Both mutant and isogenic iPSC lines were maintained on vitronectin (Thermo Fisher Scientific, MA, USA) coated plates and cultured in StemFlex medium (Thermo Fisher Scientific). A healthy donor iPSC line, CERA007c6 (2), was maintained on vitronectin-coated plates and cultured in TeSR-E8 medium (STEMCELL Technologies, Vancouver, Canada). All human iPSC lines were maintained at 37°C in a humidified atmosphere with 5% CO_2_. Culture medium was refreshed every two to three days, and cells were passaged weekly using ReLeSR (STEMCELL Technologies).

**Cardiomyocyte differentiation**

Cardiomyocytes were derived from iPSCs using previously described methods (3, 4) with modifications. iPSCs were seeded onto hESC-qualified Matrigel (Corning, NY, USA) coated plates at a density of 2.0×10^5^ cells/cm^2^ in StemFlex medium supplemented with 10 µM Y-27632 (Abcam, Cambridge, UK). After 48 hours, medium was replaced with RPMI 1640 basal medium (Thermo Fisher Scientific) containing B-27 without insulin supplement (Thermo Fisher Scientific), growth factor reduced Matrigel (1:60 dilution; Corning), and 6-8 µM CHIR99021 (STEMCELL Technologies) (designated as day 0). After 24 hours, medium was replaced with RPMI 1640 basal medium containing B-27 without insulin supplement and 5 ng/ml Activin A (Lonza, Basel, Switzerland) for 24 hours. On day 2, the medium was replaced with RPMI 1640 basal medium containing B-27 without insulin supplement and 5 µM IWP2 (Tocris Bioscience, Bristol, UK) for 72 hours. From day 5 onwards, cells were cultured in RPMI 1640 basal medium containing B-27 supplement (Thermo Fisher Scientific) and 20 ng/mL L-ascorbic acid 2-phosphate sesquimagnesium salt hydrate (Sigma-Aldrich, MA, USA) (referred to as CM medium), and the medium was refreshed every 2-3 days. On day 12, cardiomyocytes were dissociated into single cells and split at a 1:4 ratio onto hESC-qualified Matrigel coated plates in DMEM/F-12 GlutaMAX media (Thermo Fisher Scientific) supplemented with 20% fetal bovine serum (Bovogen), 100 µM 2-mercaptoethanol (Thermo Fisher Scientific), 100 µM non-essential amino acids (Thermo Fisher Scientific), 50 U/mL penicillin/streptomycin (Thermo Fisher Scientific) (referred to as CM replating medium), and 10 µM Y-27632. On day 13, the medium was replaced with CM medium. From days 14 to 19, cardiomyocytes were enriched to >95% cardiac troponin T positive cells by culture in glucose-free DMEM medium (Thermo Fisher Scientific) containing 4 mM sodium L-lactate (Sigma-Aldrich). On day 19, cardiomyocytes were dissociated and seeded at a density of 2.5×10^4^ cells/cm^2^ on hESC-qualified Matrigel coated plates in CM replating medium supplemented with 10 µM Y-27632 dihydrochloride. On days 20 and 21, the medium was changed to CM medium, and cardiomyocytes were harvested for endpoints on day 23.

**Endothelial cell differentiation**

Human iPSCs were differentiated into CD31 positive endothelial cells according to a previously published protocol (5). FA1 and FA1ic iPSCs were dissociated into single cells and seeded onto hESC-qualified Matrigel-coated plates at a density of 1×10^5^ cells/cm^2^ in StemFlex medium supplemented with 10 µM Y-27632. After 24 hours (designated as day 0), the medium was replaced with DMEM/F-12 GlutaMAX medium containing N-2 supplement (Thermo Fisher Scientific), B-27 supplement, 8 µM CHIR99021, and 25 ng/mL BMP4 (STEMCELL Technologies) for 72 hours. On day 3, the medium was replaced with complete StemPro-34 SFM medium (Thermo Fisher Scientific) supplemented with 200 ng/mL VEGF-165 (PeproTech) and 2 µM forskolin (Sigma-Aldrich), and cells were cultured for an additional 72 hours. On day 6, cells were labelled with FITC-conjugated anti-human CD31 antibody (mouse IgG; BD Pharmingen), and CD31 positive cells were isolated via fluorescence-activated cell sorting. Sorted CD31 positive endothelial cells were then expanded on plates coated with 10 µg/mL human fibronectin (Thermo Fisher Scientific) in EGM2-MV medium (Lonza) supplemented with 50 ng/mL VEGF-165 and 10 µM Y-27632 until reaching confluence (designated as passage 0).

**Smooth muscle cell differentiation**

Vascular smooth muscle cells were differentiated from human iPSCs according to a published protocol with modifications (6). FA1 and FA1ic iPSCs were dissociated and seeded onto Matrigel-coated plates at a density of 4×10^5^ cells/cm^2^ in StemFlex medium supplemented with 10 μM Y-27632. After 24 hours (designated as day 0), the medium was replaced with N2B27 medium (1:1 ratio mixture of DMEM/F-12 GlutaMAX medium and Neurobasal medium (Thermo Fisher Scientific) plus N-2 supplement and B-27 minus vitamin A supplement (Thermo Fisher Scientific)) supplemented with 8 μM CHIR99021 and 25 ng/mL BMP4. On day 3, the medium was replaced with N2B27 medium supplemented with 10 ng/mL PDGF-BB (PeproTech) and 2 ng/mL Activin A (PeproTech). On day 4, cells were replated at 8×10^5^ cells/cm^2^ onto collagen (Sigma-Aldrich) coated plates in N2B27 medium supplemented with 2 μg/mL heparin (Sigma-Aldrich) and 2 ng/mL Activin A. From day 4 to day 7, cells were maintained in this medium, after which it was replaced with SmGM-2 medium (Lonza) and continued until day 10.

**Autonomic neuron differentiation**

Autonomic neurons were derived from human iPSCs as previously described (4, 7). Briefly, FA1 and FA1ic iPSCs were seeded into ultra-low attachment round-bottom 96-well plates at a density of 2.0×10^4^ cells/well in E6 medium (Thermo Fisher Scientific) supplemented with 2 µM CHIR99021, 10 µM SB431542 (STEMCELL Technologies), and 10 µM Y-27632 (designated as day 0). On day 3, the medium was replaced by E6 medium supplemented with 20 ng/mL FGF-2 (PeproTech), 50 ng/mL BMP4, 100 nM retinoic acid (Sigma-Aldrich) and 50 U/mL penicillin/streptomycin. The medium was refreshed on day 7. On day 10, neurospheres were collected and dissociated with TrypLE™ Select Enzyme (Thermo Fisher Scientific), then re-seeded into 6-well ultra-low attachment plate (Corning) in Neurobasal Plus basal medium (Thermo Fisher Scientific) supplemented with N-2 Plus supplement (Thermo Fisher Scientific), B-27 Plus supplement (Thermo Fisher Scientific), GlutaMAX (Thermo Fisher Scientific), 20 ng/mL bFGF, 20 ng/mL EGF (PeproTech), 50 ng/mL BMP4, and 2 µg/mL heparin (Sigma-Aldrich). The medium was refreshed on day 14. Beginning on day 18, cultures were maintained in Neurobasal Plus medium supplemented with N-2 Plus supplement, B-27 Plus supplement, 10 ng/mL BDNF (PeproTech), 10 ng/mL GDNF (PeproTech), and 10 ng/mL NGF (PeproTech), with medium changes every 3-4 days to support neuronal maturation. On day 32, neurospheres were dissociated and either cryopreserved or used for the construction of cardiac microtissues.

**Cardiac fibroblast differentiation**

Cardiac fibroblasts were differentiated from iPSCs according to a previously published method (4, 8). iPSCs were dissociated into single cells and seeded onto Matrigel-coated plates at a density of 2.5×10^4^ cells/cm^2^ in StemFlex medium supplemented with 10 μM Y-27632. After 24 hours (designated as day 0), the medium was replaced with STEMdiff™ APEL™ 2 medium (STEMCELL Technologies) supplemented with 20 ng/ml BMP4, 20 ng/ml Activin A, and 1.5 µM CHIR99021 for 3 days. On day 3, the medium was replaced with STEMdiff™ APEL™ 2 medium containing 30 ng/ml BMP4, 1 µM retinoic acid, and 5 µM IWP-2. On day 6, the medium was replaced with STEMdiff™ APEL™ 2 medium containing 30 ng/ml BMP4 and 1 µM retinoic acid. On day 9, cells were dissociated and replated at a density of 1.5×10^4^ cells/cm^2^ onto human fibronectin-coated plates in STEMdiff™ APEL™ 2 medium containing 10 μM SB431542 and 10 μM Y-27632. On day 13, epicardial cells were dissociated and replated at 2.5×10^4^ cells/cm^2^ onto 0.1% porcine gelatine-coated plates and cultured in STEMdiff™ APEL™ 2 medium containing 10 ng/ml FGF-2 and 10 μM Y-27632. The medium was changed every 2 days, omitting Y-27632 after initial replating. On day 20, the medium was switched to FGM-3 medium (Lonza) and refreshed every 2-3 days thereafter.

**Iron-induced metabolic stress**

To simulate iron-induced metabolic stress conditions, 200 μM of iron sulfate was added to the CM medium from day 21 to day 23. Iron sulfate was prepared as a 100 mM stock solution by dissolving 20 mg of iron sulfate tetrahydrate (Sigma-Aldrich) in DPBS containing 0.1% of bovine serum albumin (Sigma Aldrich) adjusted to pH 5.5 (9, 10).

**Reverse transcription-quantitative polymerase chain reaction (RT-qPCR)**

Total RNA was extracted from iPSCs and cardiomyocytes using Tri-Reagent (Thermo Fisher Scientific), followed by RNA precipitation with chloroform and isopropanol (Sigma-Aldrich). cDNA was synthesized using a high-capacity cDNA reverse transcription kit (Applied Biosystems, CA, USA). Quantitative PCR was performed in duplicate using TaqMan Universal Master Mix, the QuantStudio 6 Flex Real-Time PCR system, and TaqMan Gene Expression Assays for *FXN* (Hs00175940_m1), *TNNT2* (Hs01109515_m1), *ACTC1* (Hs01109515_m1), *NANOG* (Hs04260366_g1), *SOX2* (Hs01053049_s1), *MEG3* (Hs00292028_m1), *PCDHGA10* (Hs00259371_s1), and *GAPDH* (Hs99999905_m1) (Applied Biosystems, CT, USA). Relative mRNA expression was quantified using the comparative CT method (2^-ΔΔCt^) method, normalized to *GAPDH*.

**Frataxin protein expression**

Relative frataxin protein expression was determined using the Frataxin Protein Quantity Dipstick Assay Kit (ab109881, Abcam) according to the manufacturer’s protocols. Briefly, 4 µg of total protein from iPSCs or cardiomyocytes, quantified using the Pierce™ BCA Assay (Thermo Fisher Scientific), was applied to microplate wells pre-coated with dried gold-conjugated frataxin antibody and incubated for 5 minutes at room temperature. The samples were then wicked onto dipsticks, allowed to dry at room temperature, and imaged using a flatbed scanner. Band intensities were quantified using ImageJ software.

***FXN* differential methylation region (DMR) methylation analyses**

CpG methylation analyses were performed using a protocol modified from (11). Briefly, 250 ng of genomic DNA was incubated with methylation specific restriction endonucleases HhaI (2 hours at 37°C) or BstUI (2 hours at 60°C), along the appropriate control reactions lacking the enzymes (undigested). After restriction digestion, DNA was purified by phenol-chloroform extraction and precipitation.

For methylation-sensitive PCR (MS-PCR), the DMR fragment was amplified using 1 ng of purified DNA and primers DMR_fwd 5’‐GTGTTGTGTGTGTGTGTTTG‐3’ and DMR_rev 5’‐GCTATTCTGCAGCTCTGAAAGTT‐3’. These primers amplify a region of 134 bp spanning CpG dinucleotides 63-70 of *FXN* gene (relative to the first CpG 1 located in exon 1 downstream of the ATG start codon). The HhaI and BstUI enzymes cleave unmethylated CpGs at positions 63, 64, 66, 67 and 68.

DMR methylation levels were quantified by MS-qPCR using the ΔΔCt method with Bio-Rad SsoAdvanced Universal SYBR Green Supermix. Methylation was calculated relative to an internal control PCR product lacking HhaI and BstUI cleavage sites located downstream of the DMR in the *FXN* gene. This control DNA fragment was amplified using primers DMR_CTR_for 5’-ACGGCTTGAAAGGAGGAAAC-3’ and DMR_CTR_rev 5’- TCTACCACTTCCTTGGCATC-3’.

**Immunofluorescence**

Cardiomyocytes were fixed (10% neutral buffered formalin; Trajan Scientific) for 20 minutes at room temperature and permeabilized (0.2% Triton X-100) for 10 minutes. After blocking with serum-free blocking solution (Dako, VIC, Australia) for 10 minutes, cells were stained with the specified primary antibodies overnight at 4°C, followed by secondary antibodies and 1 μg/mL of DAPI (Invitrogen) for 1 hour at room temperature.

*Cardiomyocyte hypertrophy*

To assess cardiomyocyte hypertrophy, fixed cardiomyocytes were immunostained with cardiac troponin-T (2 µg/mL, rabbit polyclonal, ab45932, Abcam) and α-actinin (25 µg/mL, mouse monoclonal, A7811, Sigma-Aldrich). The cell boundaries of cardiomyocytes positive for both cardiac troponin T and α-actinin were manually outlined, and their cell surface area was quantified using Image J software.

*Mitochondrial morphology*

Mitochondrial morphology was assessed by Hsp60 immunostaining of cardiomyocytes cultured on glass coverslips, as previously described (12, 13). Fixed cells were stained with α-actinin and Hsp60 (2 µg/mL, rabbit polyclonal, ab46798, Abcam). Coverslips were mounted onto glass slides using fluorescence mounting medium (Dako), and images were acquired using an Olympus BX61 fluorescence microscope at 600x magnification. For each replicate, images were captured from 10 randomly selected fields of view, and a minimum of 60 α-actinin-positive cardiomyocytes were analyzed. Mitochondrial morphology was classified as either predominantly networked or fragmented, based on whether more than 50% of the mitochondria in a given cell exhibited that specific morphology. For each condition, the percentages of cells classified as having networked or fragmented mitochondria were calculated, summing to 100%.

*Multicellular cardiac microtissues*

Multicellular cardiac microtissues were fixed in 10% neutral buffered formalin for 2 hours at room temperature, then dehydrated in 20% sucrose solution for 24 hours. Dehydrated samples were embedded in Optimal Cutting temperature compound (Sakura Finetek, CA, USA), and cryosectioned at 10 µm thickness. Sections were permeabilized with 0.2% Triton X-100 and blocked using Protein Block (Dako) before incubation with primary antibodies: cardiac troponin T (2 μg/mL, rabbit polyclonal, Abcam), cardiac troponin T (4 μg/mL, mouse monoclonal, ab8295, Abcam), CD31 (2 μg/mL, mouse monoclonal, M0823, Dako), and peripherin (2 μg/mL, mouse monoclonal, sc377093, Santa Cruz). Following primary antibody incubation, sections were stained with secondary antibodies: Alexa Fluor 488-conjugated goat-anti-rabbit or goat-anti-mouse (10 μg/mL, A11001/A11008, Invitrogen), and Alexa Fluor 594-conjugated goat-anti-mouse or goat-anti-rabbit (10 μg/mL, A11005/A11012, Invitrogen). Nuclei were counterstained with 1 μg/mL of DAPI (Invitrogen). Epifluorescence images were acquired using an Olympus BX61 upright microscope and analySIS software.

**Contraction analysis**

Cardiomyocyte and cardiac microtissue contractions were recorded using an inverted Olympus IX-71 microscope (4, 14). Videos were recorded at either 30 frames per second (for cardiac microtissues) or 60 frames per second (for 2D cardiomyocytes) for 15 seconds or until at least ten complete contraction cycles were captured. Contraction analysis was performed using the MUSCLEMOTION plugin (15) in ImageJ software, with the following setting: frames/second (30 or 60), speedWindow (2), default noise reduction, automatic reference frame, and automatic peak fitting. Beat-to-beat variability was quantified by calculating the root mean square of successive differences (RMSSD) from 10 successive beats using the formula: $\sqrt{\frac{\sum_{i=1}^{N-1} {({RR}_{i}-{RR}_{i+1})}^{2}}{N-1}}$, where N is the number of beats, and RR represents the interval between adjacent contraction peaks (16).

**Field potential duration**

Cardiomyocyte electrophysiological activity was evaluated using a multielectrode array (MEA) recording system (Multichannel Systems, Reutlingen, Germany), as previously described (17). Day 19 cardiomyocytes were seeded onto MEA plates pre-coated with hESC-qualified Matrigel and cultured for 48 hours. Extracellular field potentials were recorded for 5 minutes, and data were analyzed offline using MC Rack software (version 4.3.5) to determine RR intervals and extracellular field potential duration (FPD). FPD values were normalized using Fridericia’s correction formula: corrected FPD = FPD/(RR interval)^1/3^.

**Calcium transient analysis**

Calcium transient amplitude and regularity were assessed using the calcium-sensitive indicator Fluo-4 acetoxymethyl ester (Thermo Fisher Scientific). Fluo-4 was prepared at a final concentration of 1 μM in Hanks’ Buffer Saline Solution with Mg^2+^ and Ca^2+^ (HBSS++, Thermo Fisher Scientific). Cardiomyocytes were incubated with Fluo-4 at 37°C in a humidified CO_2_ incubator, protected from light, for 20 minutes. After incubation, cells were washed twice with HBSS++ and imaged immediately in fresh HBSS++ using an inverted Olympus IX-71 microscope. Calcium transients were analyzed by plotting corrected total cell fluorescence of Fluo-4 signal over time. Peak amplitude was quantified as the change in fluorescence intensity relative to baseline (Δ(F-F_0_)) across three consecutive peaks. Calcium traces displaying abnormal spike morphology or irregular rhythmicity were classified as irregular, based on published criteria (18, 19), and expressed as a percentage of total cardiomyocytes analyzed. For each biological replicate, calcium transients were recorded from 20 cardiomyocytes sampled from at least 3 random fields.

**Cell death assay**

Cardiomyocyte viability was assessed using propidium iodide and Hoechst 33258 staining. Cells were incubated with 5 μg/mL of propidium iodide and 1 μg/mL of Hoechst 33258 (Sigma-Aldrich), prepared in HBSS++, for 45 minutes at 37°C in a humidified CO_2_ incubator, protected from light. Fluorescent images were captured using an inverted Olympus IX-71 microscope. The number of dead cells (propidium iodide positive) was counted and expressed as a percentage of the total number of cells (Hoechst 33258 positive). At least 100 cells per condition were analyzed in each independent experiment.

**Mitochondrial ROS measurement**

Mitochondrial ROS levels in cardiomyocytes were assessed using the MitoSOX™ Red dye (Thermo Fisher Scientific). Cells were incubated with 5 μM MitoSOX™ Red in HBSS++ for 20 minutes at 37°C in a humidified CO_2_ incubator, protected from light. Following incubation, cells were washed once with HBSS++ and imaged immediately in fresh HBSS++ using an inverted Olympus IX-71 microscope at 200x magnification. The corrected total cell fluorescence was quantified for 100 cardiomyocytes from 3 random fields per group in each independent experiment.

**Mitochondrial membrane potential measurement**

Mitochondrial membrane potential was assessed using tetramethylrhodamine methyl ester (TMRM, Thermo Fisher Scientific). Cardiomyocytes were incubated with 10 nM TMRM in HBSS++ (non-quenching concentration) for 20 minutes at 37°C in a humidified CO_2_ incubator, protected from light. Imaging was performed immediately after incubation, without a wash step, using an inverted Olympus IX-71 microscope at 200x magnification. The corrected total cell fluorescence was quantified for 100 cardiomyocytes across 3 random fields for each group in each independent experiment.

**Iron assay**

Cellular total iron levels were quantified using the Iron Assay Kit (ScienCell Research Laboratories, CA, USA) in accordance with the manufacturer’s instructions. Briefly, frozen cell pellets were resuspended in the provided working buffer and homogenised using the Bead Mill Tissue Homogenizer (Tissue Lyser LT, Qiagen, Venlo, Netherlands). The resulting lysates were incubated with the working reagent, and absorbance was measured at 590 nm using a microplate reader. Iron concentrations were calculated from a standard curve and normalized to total protein content, which was determined using the Pierce™ BCA Protein Assay Kit (Thermo Fisher Scientific, USA).

***MEG3* and *PCDHGA10* knockdown in cardiomyocytes**

Day 19 cardiomycoytes derived from FA1 and FA1ic iPSCs were seeded at 2.5×10⁴ cells/cm^2^ onto Matrigel-coated plates in cardiomyocyte maintenance medium and allowed to recover for 48 hours. Transfections were performed using Lipofectamine^®^ RNAiMAX (Thermo Fisher Scientific), diluted to 0.15x the manufacturer’s recommended concentration in Opti MEM^®^ (Gibco) and incubated at room temperature for 15 minutes to allow complex formation. For *MEG3* knockdown, an LNA™ GapmeR antisense oligonucleotide targeting *MEG3* (Qiagen; NR_002766.2_1; GeneGlobe ID LG00818663-DDA; Cat# 339512) was used at a final concentration of 50 nM, together with a scrambled GapmeR control (50 nM; Negative Control A; Qiagen; GeneGlobe ID LG00000002-DDA; Cat#339516). For *PCDHGA10* knockdown, a SMARTPool siRNA targeting *PCDHGA10* (Dharmacon; Cat# L-013294-02) was used at a final concentration of 10 nM, together with a non-targeting scrambled SMARTPool siRNA control (10 nM; Dharmacon; Cat# D-001810-10). Cells were exposed to transfection complexes for 24 hours, after which the medium was replaced, and cells were maintained for a further 48 hours prior to endpoint assays.

**RNA sequencing**

Total RNA was extracted from cardiomyocytes derived from all three FRDA and isogenic control iPSC lines across three independent experiments using the Cytivia Mini RNA Extraction Kit (Cytivia, MA, USA), following the manufacturer’s instructions. Residual genomic DNA was removed by treatment with TURBO DNase (Thermo Fisher Scientific). RNA integrity was assessed using the Agilent 2100 Bioanalyzer (Agilent Technologies), and all samples exhibited an RNA Integrity Number (RIN) of 10.

Library preparation was performed using 100 ng of input RNA per sample with the TruSeq Stranded Total RNA Library Prep Kit (poly‑A mRNA enrichment via oligo‑dT magnetic beads; Illumina, CA, USA) as per manufacturer’s instruction. The library was quantified using the Agilent Tapestation and the Qubit™ RNA assay kit for Qubit 2.0® Fluorometer (Life technologies). Unique dual indexes (Illumina TruSeq RNA UD Indexes) were used to barcode individual libraries. The indexed libraries were then prepared and diluted to 750pM for paired end (2x 101 base) sequencing on a NextSeq2000 instrument using the P3 200 cycle kit using v3 chemistry (Illumina, CA, USA) as per manufacturer’s instructions. The base calling and quality scoring were determined using Real-Time Analysis on board software v2.4.6, while the FASTQ file generation and de-multiplexing utilised bcl2fastq conversion software v2.15.0.4. Libraries yielded a median of 26.95 million read pairs per sample, with a median Q30 score of 95.6% and a median Salmon mapping rate of 91.0%.

RNA sequencing (RNA-seq) data were processed and analyzed using the ARMOR (Automated Reproducible Modular Workflow for Preprocessing and Differential Analysis of RNA-seq Data) pipeline (20). Following read quality assessment and adapter trimming, adapters and low‑quality bases were removed with Trim Galore! (‑q 20). Transcript abundance was quantified using Salmon with quasi‑mapping to the Ensembl GRCh38 (release 93) transcriptome, with sequence‑ and GC‑bias correction enabled; gene‑level counts were imported using tximeta/tximport. For differential gene expression analysis, lowly expressed genes were filtered, library sizes were normalized using TMM, and edgeR was used to estimate dispersions and fit quasi‑likelihood negative binomial generalized linear models to assess differential expression between experimental conditions (21). Genes with a false discovery rate (FDR) below a specified threshold (FDR < 0.05) were considered significantly differentially expressed. All analyses were performed with default parameters unless otherwise specified, ensuring consistency and reproducibility across samples.

Pathway enrichment analysis was performed using the PANTHER Overrepresentation Test (release date: 2024-08-07) via the PANTHER online platform (22) and visualised using SRplot (23). Annotations were based on the GO Ontology database [DOI: 10.5281/zenodo.15066566], released on 2025-03-16. The input gene list included all differentially expressed genes (DEGs) with an FDR < 0.05 and an absolute log fold change > 2. The reference background included all identified DEGs. Enrichment was assessed using Fisher’s Exact Test, and multiple testing correction was applied using the FDR method.

**Supplementary Figure 1. Expression of pluripotency and cardiomyocyte markers in FRDA and isogenic control iPSCs and iPSC‑derived cardiomyocytes.** (A-D) mRNA expression of NANOG (A), SOX2 (B), TNNT2 (C), and ACTC1 (D) in FRDA iPSCs (orange bars), isogenic control iPSCs (blue bars), FRDA iPSC-derived cardiomyocytes (orange patterned bars), and isogenic control iPSC-derived cardiomyocytes (blue patterned bars). n = 3-4 independent experiments. Data are shown for individual iPSC/cardiomyocyte pairs (FA1, FA2, FA3) and as a pooled analysis across all three lines (FA1-3). Data were analysed by two‑way ANOVA (factors: cell type and genotype), followed by simple main‑effects comparisons of FRDA versus isogenic controls where interactions were significant. *p<0.05, **p<0.01, ***p<0.001, ****p<0.0001.

**Supplementary Figure 2. Excision of the GAA repeats reduces hypermethylation of the FRDA‐related differentially methylated regions (DMRs) in isogenic control iPSCs to levels comparable to unaffected control iPSCs.** (A) Results of methylation‐specific PCR (MS-PCR) and (B) methylation-specific qPCR (MS-qPCR) performed using DNA isolated from iPSCs are shown. FA2 and its isogenic derivative FA2ic, lacking GAA repeats, were used in analyses along with unaffected control cells (CTR, short GAA repeats). Two different CpG methylation sensitive restriction enzymes (HhaI and BstUI) were used. “Und” indicates a control PCR reaction performed using undigested DNA, “-” designates a no-DNA template control reaction, and “L” indicates a 100 bp DNA ladder. Real-time PCR quantitation was performed relative to a DNA fragment of the FXN gene lacking HhaI and BstUI recognition sites.

**Supplementary Figure 3. Extracellular field potential of Friedreich ataxia iPSC-derived cardiomyocytes.** (A-B) Representative trace of extracellular field potential of FA1 (A) and FA1ic (B) cardiomyocytes recorded from multielectrode array. (C-D) Field potential duration (C) and corrected field potential duration (D) of FA1 and FA1ic cardiomyocytes (n = 4 independent experiments).

**Supplementary Figure 4. Cell death in FRDA iPSC-derived cardiomyocytes is exacerbated by iron supplementation.** Representative images showing cell death detected by propidium iodide (PI; red) and Hoechst (blue) co-staining in cardiomyocytes derived from FRDA (FA1) and isogenic control (FA1ic) iPSCs. Cells were maintained under basal conditions (Basal) or exposed to 200 µM iron sulphate for 48 hours (+iron). Red arrows indicate PI-positive nuclei. Scale bar = 100 µm.

**Supplementary Figure 5. Representative cell death images following *PCDHGA10* or *MEG3* knockdown in FRDA iPSC‑derived cardiomyocytes.** (A-B) Representative images showing cell death detected by propidium iodide (PI; red) and Hoechst (blue) staining in isogenic control and FRDA iPSC‑derived cardiomyocytes treated with (A) scrambled siRNA or *PCDHGA10*‑targeting siRNA SMARTpool, or (B) scrambled antisense oligonucleotide (ASO) or *MEG3*‑targeting ASO. Red arrows indicate PI‑positive nuclei. Scale bar = 100 µm.

**Supplementary Table Legends**

**Supplementary Table S1.** Differential expression analysis results for cardiomyocytes from patient 1 (FA1), generated using edgeR within the ARMOR pipeline.

**Supplementary Table S2.** Differential expression analysis results for cardiomyocytes from patient 2 (FA2), generated using edgeR within the ARMOR pipeline.

**Supplementary Table S3.** Differential expression analysis results for cardiomyocytes from patient 3 (FA3), generated using edgeR within the ARMOR pipeline.

**Supplementary Table S4.** Differential expression analysis results from the combined analysis of cardiomyocytes from patients 1-3 (FA1-FA3), generated using edgeR within the ARMOR pipeline.

**Supplementary Table S5.** GO Biological Process enrichment from PANTHER Overrepresentation Test on combined patient cardiomyocyte data (FDR < 0.05, |Fold Change| ≥ 2).

**References**

1.	Li Y, Li J, Wang J, Zhang S, Giles K, Prakash TP, et al. Premature transcription termination at the expanded GAA repeats and aberrant alternative polyadenylation contributes to the Frataxin transcriptional deficit in Friedreich's ataxia. Hum Mol Genet. 2022;31(20):3539–57.

2.	Hernández D, Millard R, Sivakumaran P, Wong RC, Crombie DE, Hewitt AW, et al. Electrical Stimulation Promotes Cardiac Differentiation of Human Induced Pluripotent Stem Cells. Stem Cells Int. 2016;2016:1718041.

3.	Lyu Q, Gong S, Lees JG, Yin J, Yap LW, Kong AM, et al. A soft and ultrasensitive force sensing diaphragm for probing cardiac organoids instantaneously and wirelessly. Nat Commun. 2022;13(1):7259.

4.	Lees JG, Greening DW, Rudd DA, Cross J, Rosdah AA, Lai X, et al. Cardiac-targeted delivery of a novel Drp1 inhibitor for acute cardioprotection. J Mol Cell Cardiol Plus. 2024;9:100085.

5.	Kong AM, Yap KK, Lim SY, Marre D, Pébay A, Gerrand YW, et al. Bio-engineering a tissue flap utilizing a porous scaffold incorporating a human induced pluripotent stem cell-derived endothelial cell capillary network connected to a vascular pedicle. Acta Biomater. 2019;94:281–94.

6.	Patsch C, Challet-Meylan L, Thoma EC, Urich E, Heckel T, O'Sullivan JF, et al. Generation of vascular endothelial and smooth muscle cells from human pluripotent stem cells. Nat Cell Biol. 2015;17(8):994–1003.

7.	Kirino K, Nakahata T, Taguchi T, Saito MK. Efficient derivation of sympathetic neurons from human pluripotent stem cells with a defined condition. Sci Rep. 2018;8(1):12865.

8.	Giacomelli E, Meraviglia V, Campostrini G, Cochrane A, Cao X, van Helden RWJ, et al. Human-iPSC-Derived Cardiac Stromal Cells Enhance Maturation in 3D Cardiac Microtissues and Reveal Non-cardiomyocyte Contributions to Heart Disease. Cell Stem Cell. 2020;26(6):862–79.e11.

9.	Lee YK, Ho PW, Schick R, Lau YM, Lai WH, Zhou T, et al. Modeling of Friedreich ataxia-related iron overloading cardiomyopathy using patient-specific-induced pluripotent stem cells. Pflugers Arch. 2014;466(9):1831–44.

10.	Lee YK, Lau YM, Ng KM, Lai WH, Ho SL, Tse HF, et al. Efficient attenuation of Friedreich's ataxia (FRDA) cardiomyopathy by modulation of iron homeostasis-human induced pluripotent stem cell (hiPSC) as a drug screening platform for FRDA. Int J Cardiol. 2016;203:964–71.

11.	Rodden LN, Chutake YK, Gilliam K, Lam C, Soragni E, Hauser L, et al. Methylated and unmethylated epialleles support variegated epigenetic silencing in Friedreich ataxia. Hum Mol Genet. 2021;29(23):3818–29.

12.	Hoque A, Sivakumaran P, Bond ST, Ling NXY, Kong AM, Scott JW, et al. Mitochondrial fission protein Drp1 inhibition promotes cardiac mesodermal differentiation of human pluripotent stem cells. Cell Death Discov. 2018;4:39.

13.	Rosdah AA, Abbott BM, Langendorf CG, Deng Y, Truong JQ, Waddell HMM, et al. A novel small molecule inhibitor of human Drp1. Sci Rep. 2022;12(1):21531.

14.	Claridge B, Rai A, Lees JG, Fang H, Lim SY, Greening DW. Cardiomyocyte intercellular signalling increases oxidative stress and reprograms the global- and phospho-proteome of cardiac fibroblasts. J Extracell Biol. 2023;2(12):e125.

15.	Sala L, van Meer BJ, Tertoolen LGJ, Bakkers J, Bellin M, Davis RP, et al. MUSCLEMOTION: A Versatile Open Software Tool to Quantify Cardiomyocyte and Cardiac Muscle Contraction In Vitro and In Vivo. Circulation Research. 2018;122(3):e5–e16.

16.	Yin J, Lees JG, Gong S, Nguyen JT, Phang RJ, Shi Q, et al. Real-time electro-mechanical profiling of dynamically beating human cardiac organoids by coupling resistive skins with microelectrode arrays. Biosens Bioelectron. 2025;267:116752.

17.	Lees JG, Kong AM, Chen YC, Sivakumaran P, Hernández D, Pébay A, et al. Mitochondrial Fusion by M1 Promotes Embryoid Body Cardiac Differentiation of Human Pluripotent Stem Cells. Stem Cells Int. 2019;2019:6380135.

18.	Gaballah M, Penttinen K, Kreutzer J, Mäki AJ, Kallio P, Aalto-Setälä K. Cardiac Ischemia On-a-Chip: Antiarrhythmic Effect of Levosimendan on Ischemic Human-Induced Pluripotent Stem Cell-Derived Cardiomyocytes. Cells. 2022;11(6).

19.	Hwang H, Liu R, Maxwell JT, Yang J, Xu C. Machine learning identifies abnormal Ca(2+) transients in human induced pluripotent stem cell-derived cardiomyocytes. Sci Rep. 2020;10(1):16977.

20.	Orjuela S, Huang R, Hembach KM, Robinson MD, Soneson C. ARMOR: An Automated Reproducible MOdular Workflow for Preprocessing and Differential Analysis of RNA-seq Data. G3 (Bethesda). 2019;9(7):2089–96.

21.	Chen Y, Chen L, Lun ATL, Baldoni PL, Smyth GK. edgeR v4: powerful differential analysis of sequencing data with expanded functionality and improved support for small counts and larger datasets. Nucleic Acids Res. 2025;53(2).

22.	Mi H, Muruganujan A, Huang X, Ebert D, Mills C, Guo X, et al. Protocol Update for large-scale genome and gene function analysis with the PANTHER classification system (v.14.0). Nat Protoc. 2019;14(3):703–21.

23.	Tang D, Chen M, Huang X, Zhang G, Zeng L, Zhang G, et al. SRplot: A free online platform for data visualization and graphing. PLoS One. 2023;18(11):e0294236.
